# Amplicon sequencing reveals host-associated lineages of *Cystobasidiomycetes* (*Basidiomycota*, *Pucciniomycotina*) in *Arthoniales* (*Ascomycota*, *Arthoniomycetes*), but no association with thallus architecture

**DOI:** 10.3897/imafungus.17.201170

**Published:** 2026-10-21

**Authors:** Luca Borgato, Pieter Asselman, Damien Ertz, Mario Amalfi, Annemieke Verbeken

**Affiliations:** 1 Ghent University, Biology Department, Research Group Mycology, K.L. Ledeganckstraat 35, L10, 9000-Ghent, Belgium Ghent University, Biology Department, Research Group Mycology Ghent Belgium; 2 Meise Botanic Garden, Research Department, Nieuwelaan 38, 1860 Meise, Belgium Meise Botanic Garden, Research Department Meise Belgium; 3 Fédération Wallonie-Bruxelles, Service général de l’Enseignement supérieur et de la Recherche scientifique, rue A. Lavallée 1, 1080 Bruxelles, Belgium Fédération Wallonie-Bruxelles, Service général de l’Enseignement supérieur et de la Recherche scientifique Bruxelles Belgium

**Keywords:** Cortex, diversity, endolichenic fungi, ITS, MinION, selectivity, variation partitioning

## Abstract

Lichen-associated *Cystobasidiomycetes* have been reported from a wide range of lichens worldwide. Previous studies proposed that lichen-associated *Cystobasidiomycetes* may be involved in the development of the thallus cortex, suggesting also a contribution to the construction of thallus architecture within the *Parmeliaceae*. However, *Parmeliaceae* do not include species with ecorticate thalli. In contrast, *Arthoniales* encompass a wide diversity of thallus growth forms, including both corticate and ecorticate types, making this order a suitable model to test this hypothesis across a broader evolutionary context. Furthermore, *Cystobasidiomycetes* have not yet been studied in *Arthoniales*. This study aims therefore to (1) characterize the diversity of *Arthoniales*-associated *Cystobasidiomycetes*; (2) investigate their selectivity with respect to mycobiont partners and the ecological factors shaping their distribution, and (3) test whether their distribution is consistent with a proposed role in cortex formation and thallus architecture in *Arthoniales*. The ITS region of *Cystobasidiomycetes* was sequenced from 169 lichen samples (in majority *Arthoniales*) and an exhaustive maximum-likelihood phylogeny, including our sequences and all sequences found in GenBank, was inferred using IQ-tree. The effects of substratum, climate, host taxonomy and geographic location on the distribution of *Cystobasidiomycetes* were assessed using variation partitioning. *Cystobasidiomycetes* were found in 60 of our samples. Our sequences belonged to 39 *Cystobasidiomycetes* OTUs, 21 of which were detected in *Arthoniales* thalli, and eight of them represent unknown lineages. Globally, OTU selectivity was variable. However, most OTU’s exhibited a preference for particular groups of mycobionts. Mycobiont taxonomy emerged as the primary factor shaping lichen-derived *Cystobasidiomycetes* community composition. Climate and substrate likely also influence their distribution, although their effects may be underestimated, due to an unbalanced dataset, dominated by sequences from extra-tropical and corticolous specimens. Finally, no evidence was found to support a correlation between *Cystobasidiomycetes* detection, thallus growth form or cortex presence in *Arthoniales*.

Overall, these results suggest that lichen-derived *Cystobasidiomycetes* OTUs largely form lineages distinct from those represented by *Cystobasidiomycetes* from other sources. However, the lifestyle of many sequences remains uncertain because they were obtained through environmental sequencing. Despite their generally low selectivity, a consistent pattern of mycobiont preference was observed across *Cystobasidiomycetes* orders. This pattern was not associated with thallus growth form or the presence of a cortex.

## Introduction

### Lichen-associated fungi

Traditionally, a lichen is defined as a symbiotic association between a primary fungus (the mycobiont or lichenized fungus) and a photosynthetic partner (the photobiont; e.g., Frank 1877; Schwendener 1869, 1873), such as a cyanobacterium or an alga (Leliaert et al. 2012; Magain and Sérusiaux 2014). However, several researches have revealed that lichens are far more complex. They are now seen as a “greenhouse” (Grube 2021), “a self-sustaining ecosystem formed by the interaction of an exhabitant fungus (i.e. the mycobiont) and an extracellular arrangement of one or more photosynthetic partners (obligate symbionts) and an indeterminate number of other microscopic organisms” (Hawksworth and Grube 2020), including secondary fungi, bacteria, protists, and viruses (e.g., Millanes et al. 2016, 2025; Aschenbrenner et al. 2016; Spribille et al. 2016; Tuovinen et al. 2019, 2021; Merges et al. 2021; Acosta et al. 2024; Hakobjanyan et al. 2025). Those microorganisms may be obligate or facultative symbionts (i.e. not fundamental for the lichenic symbiosis and not necessarily associating with the host), or simply commensals. This makes lichens comparable to other obligate complex symbioses, such as coral reefs, which also host algae and a microbiome (e.g. Rosenberg et al. 2007; Maire et al. 2021).

Lichen-associated fungi are cosmopolitan and occur across all ecosystems (e.g. Stone et al. 2000; Davis et al. 2003; Arnold 2007; Arnold et al. 2009). They are generally classified as lichenicolous and endolichenic, both of which are part of the lichen microbiome (Cometto et al. 2022). While lichenicolous fungi are parasitic, showing visible epithallic symptoms and often morphological alterations in the lichen thallus (e.g., Hawksworth 1982; Rambold and Triebel 1992; Lawrey and Diederich 2003, 2011, 2016; Muggia et al. 2015; Diederich et al. 2018, 2022a, 2022b, 2024), endolichenic fungi do not produce any visible symptoms on the lichen thallus. Some lichen-associated fungi can be both lichenicolous and endolichenic (e.g. Spribille et al. 2016; Diederich et al. 2022b; Millanes et al. 2025). The role of lichen-associated fungi in the lichen microbiome is still poorly understood, as their study started only recently. This is especially the case of endolichenic fungi, which are often detected solely by microscopy, cultures or environmental DNA sequencing techniques (e.g., Arnold et al. 2009; Muggia et al. 2015, 2016, 2017, 2019; Fernández‐Mendoza et al. 2017; Tagirdzhanova et al. 2021, 2024; Cometto et al. 2022, 2023, 2024; Stoppiello et al. 2025), though some studies provided direct observation images of the latter (e.g. Spribille et al. 2016; Černajová and Škaloud 2019; Nguyen et al. 2023). Lichen-associated fungi may form mutualistic or parasitic symbioses with lichenized fungi or with photobionts (e.g., Spribille et al. 2016; Muggia and Grube 2018; Tagirdzhanova et al. 2021, 2024; Tuovinen et al. 2021; Nguyen et al. 2023; Kirkhus et al. 2025). Other endolichenic fungi simply live occasionally in the lichen thallus as commensals (e.g., Arnold et al. 2009; Muggia and Grube 2010; U’Ren et al. 2012, 2014; Banchi et al. 2018). This is particularly true for corticolous lichens, which are often found coexisting with the mycelia of various moulds, endophytes, or phytopathogenic fungi that colonize the bark (Arnold et al. 2009; U’Ren et al. 2014).

### Cystobasidiomycetes

One fungal class including lichen-associated fungi is the *Cystobasidiomycetes*, believed to have originated during the Carboniferous period (He et al. 2022). These fungi are mostly known from the yeast state (Schoutteten et al. 2024) and have colonized a wide range of habitats, from aquatic to terrestrial ecosystems. They can be found on diverse substrates including air, water, soil, rocks, bark, leaves or artificial substrates, and on various hosts, from plants (herbs as well as trees) to animals (including humans), as well as fungi and lichens (e.g., Zhao et al. 2002; Libkind et al. 2010; Spribille et al. 2016; Oberwinkler 2017; Mark et al. 2020; Nagahama et al. 2001, 2003; Nguyen et al. 2023; Schoutteten et al. 2024). The class *Cystobasidiomycetes* comprises 125 accepted species across 21 genera, eight families, and six orders (*Buckleyzymales*, *Cyphobasidiales*, *Cystobasidiales*, *Erythrobasidiales*, *Naohideales* and *Sakaguchiales*) or uncertain positions (*Microsporomycetaceae* and *Symmetrosporaceae*; Index Fungorum: https://www.indexfungorum.org/).

The first lichen-associated *Cystobasidiomycetes* to be formally recognized were *Cyphobasidium hypogymniicola* and *C. usneicola* (Diederich, 1996; as *Cystobasidium*), of the order *Cyphobasidiales* (Millanes et al. 2016; Spribille et al. 2016), found on *Hypogymnia physodes* and *Usnea* sp respectively. These fungi develop within the host thallus and can be both lichenicolous, inducing visible symptoms (galls) and (more often) endolichenic, without showing any symptom (Spribille et al. 2016; Diederich et al. 2022b). Since their initial discovery, additional lichen-associated *Cystobasidiomycetes* have been found on a wide range of lichens worldwide, including Africa (Spribille et al. 2016), the Arctic and Antarctic regions (Timling et al. 2014; Park et al. 2015; Zhang et al. 2016; Stoppiello et al. 2025), Asia (Nguyen et al. 2023), Europe (Zhang et al. 2016; Kachalkin et al. 2017, 2024; Černajová and Škaloud 2019; Mark et al. 2020), North America (Nash III et al. 2004; Millanes et al. 2016; Spribille et al. 2016; Lendemer et al. 2019; Tagirdzhanova et al. 2021) and South America (Pérez et al. 2024). Those belonged not only to the order *Cyphobasidiales*, but also to *Microsporomycetaceae* (e.g., Černajová and Škaloud 2019; Nguyen et al. 2023; Kachalkin et al. 2024; Millanes et al. 2025), *Buckleyzymales* (Mark et al. 2020) and *Cystobasidiales* (e.g., Pérez et al. 2024). However, unlike the latter three, which include species with other ecological associations, *Cyphobasidiales* are exclusively lichen-associated fungi.

Lichen-associated *Cystobasidiomycetes* gained considerable attention with the study of Spribille et al. (2016), which demonstrated that *Cyphobasidiales* may influence secondary metabolite production in *Bryoria fremontii* and proposed their involvement in thallus cortex formation in *Parmeliaceae*, since they were mostly detected in the thallus cortex of the studied specimens. This suggests that they may also contribute to the development of foliose macrolichens, particularly within the *Parmeliaceae* family, and eventually even in complex thalli of other groups. However, this hypothesis remains unconfirmed, as conversely, a later study on the genus *Cladonia* (Černajová and Škaloud 2019) demonstrated that *Cystobasidiomycetes* yeasts occur in both corticate and ecorticate species, suggesting that their presence is not strictly linked to cortex formation. Because that study was restricted to a single genus, it however remained unclear whether this pattern was general across morphologically and phylogenetically diverse lichen lineages. Moreover, the role of these endolichenic fungi as true mutualistic partners is still unclear. Some studies have concluded that they are parasites or commensals rather than core constituents of the lichen symbioses having the same status as photobionts (Oberwinkler 2017; Lendemer et al. 2019; Mark et al. 2020). These yeasts also appear to exhibit a lower degree of selectivity in mycobiont choice compared to their photobiont partners (Mark et al. 2020) and are not necessarily detected in every thallus (Lendemer et al. 2019; Cometto et al. 2022, 2024). Furthermore, their presence is not necessarily ubiquitous across lichen communities, even in species where they were found. While some studies found those yeasts to be notably sparse (e.g., Černajová and Škaloud 2019; Kachalkin et al. 2024), others, in contrast, found their occurrence more restricted (e.g., Lendemer et al. 2019; Nguyen et al. 2023). However, the methodology used to detect them may also influence the results. Depending on the sequencing approach, some low abundant fungi may remain undetected.

Research on lichen-associated *Cystobasidiomycetes* has been so far primarily focused on lichenized fungi from the order *Lecanorales* and very few others (e.g., Spribille et al. 2016; Zhang et al. 2016; Mark et al. 2020; Nguyen et al. 2023; Pérez et al. 2024), but these were never deeply studied in *Arthoniales*. Currently, only two *Cystobasidiomycetes* sequences from *Arthoniales* lichens (*Enterographa pallidella* and *Opegrapha vulgata*) are available on GenBank (Lendemer et al. 2019; Diederich et al. 2022b).

Contrarily to the family *Parmeliaceae*, *Arthoniales* show a large thallus variation, including not only species with either crustose or fruticose thalli, but also with either corticate or ecorticate thalli. The fruticose growth form and the presence of a cortex appeared or were lost several times during the evolution of the group (even in one single genus; Ertz and Tehler 2011; Frisch et al. 2014; Ertz et al. 2015), crustose ecorticate thalli being the ancestral state (Ertz et al. 2015). This order is therefore a good model, as it allows testing whether *Cystobasidiomycetes* are implicated in the cortex development, as suggested by Spribille et al. (2016) for *Parmeliaceae*, and if they are possibly involved in the formation of fruticose thalli in this order. This cannot be tested in *Parmeliaceae* (Spribille et al. 2016), because this family does not include lichenized species with ecorticate thalli. Moreover, while the study of Černajová and Škaloud (2019) was restricted to a single genus, our study involves an entire order (*Arthoniales*), which provides an ideal system to further test this hypothesis across a broader evolutionary context.

This study will (1) investigate the occurrence and degree of selectivity in the mycobiont choice of lichen-associated *Cystobasidiomycetes*, (2) unravel their possible role in the cortex architecture and the appearance of fruticose thalli in the *Arthoniales* order (3) and evaluate ecological factors influencing their distribution.

## Material and methods

### Sampling localities and material collection

**Figure 1.**
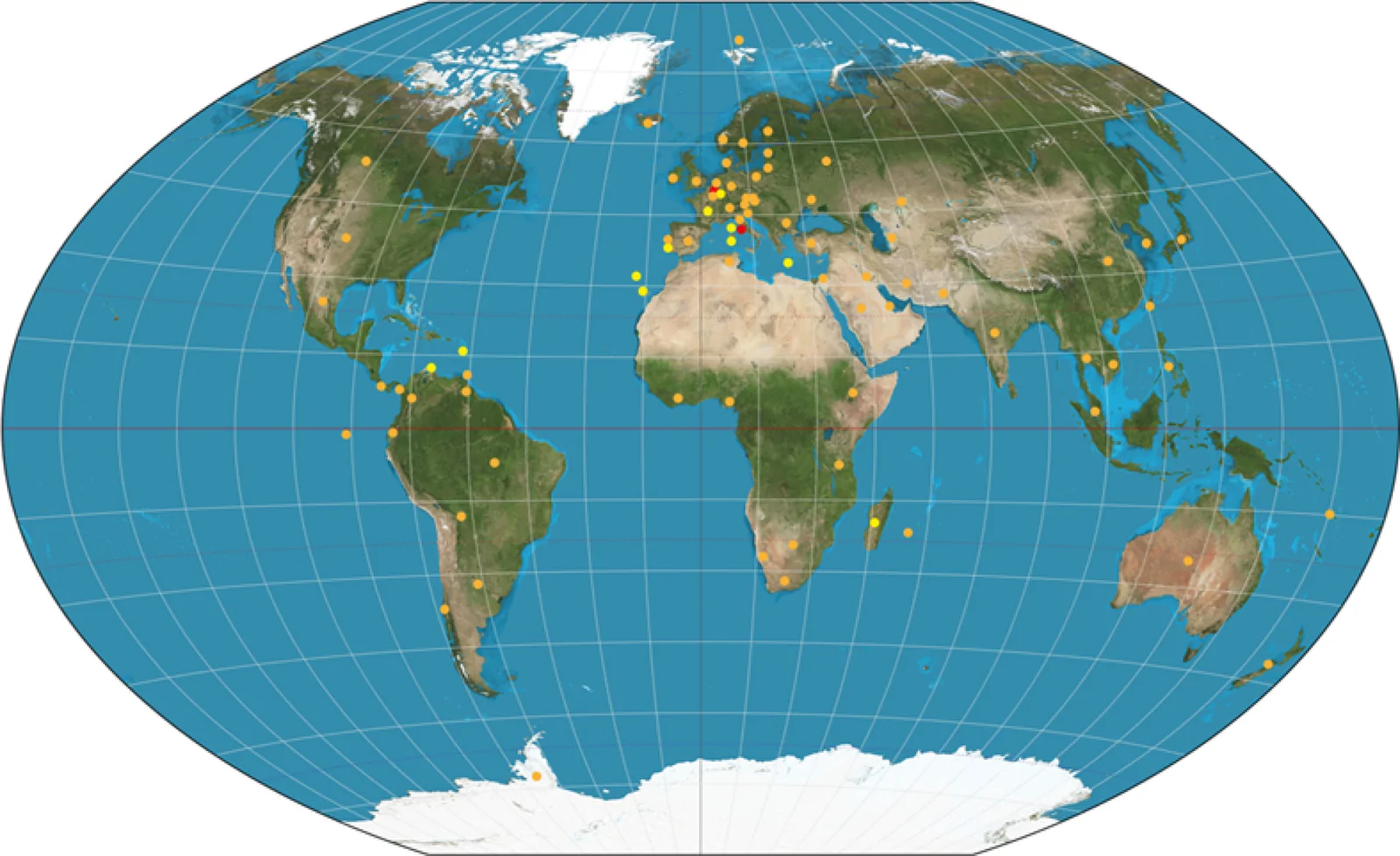
Map showing the origin of specimens used in this study (modified from Daniel R. Strebe: https://en.wikipedia.org/wiki/World_map). Red dots = locations of specimens collected in this study and stored at BR; yellow dots = locations of specimens collected in previous excursions and stored at BR; orange dots = locations of *Cystobasidiomycetes* sequences retrieved from GenBank. Note: the proportions are not showed in this map; to check the specimen’s quantity per country, see Suppl. material 4: tables S1, S3).

Lichens specimens belonging mainly to the different families of the class *Arthoniomycetes*, together with a smaller number of specimens from other classes were collected in Belgium and Italy, during 2022 and 2023 (Fig. 1, Suppl. material 4: table S1). Fresh samples were stored at the Meise Botanic Garden (BR), at – 20 °C or used directly for the molecular analyses. To expand the geographic representativeness of the dataset, additional lichen specimens previously stored in the BR collection freezers were included from France, Portugal, Crete, Madeira, Canary Islands, Cabo Verde, Madagascar, Guadeloupe or Curaçao (Fig. 1, Suppl. material 4: table S1), resulting in a total of 169 samples, including 133 *Arthoniomycetes* specimens and 36 specimens from other classes.

The selection was designed to encompass crustose, foliose (restricted to *Lecanoromycetes*), and fruticose thalli, with or without a cortex, growing either on bark (corticolous) or on rock (saxicolous). These collections enabled comparisons of *Cystobasidiomycetes* associated with *Arthoniales* to those linked with other mycobiont orders. They also allowed us to assess whether *Cystobasidiomycetes* are ubiquitous across all specimens of the species in which they have been reported (Spribille et al. 2016; Lendemer et al. 2019), as well as to investigate potential geographic variation between European specimens and those from other continents. Despite the risk to accidentally detect airborne spores or epithallic *Cystobasidiomycetes* not linked with lichens, our thalli were not cleaned, because of the risk of accidentally remove target *Cystobasidiomycetes*, especially in lichens with very thin thalli, like *Opegrapha*’s. All specimens were later deposited in the BR herbarium (Suppl. material 4: table S1).

### Molecular methods

Total DNA was extracted from the samples following the CTAB protocol (Guzow-Krzemińska and Węgrzyn 2000). The DNA concentration was quantified by using a Qubit® 2.0 fluorometer and samples were subsequently diluted to 1 ng/µl.

For each DNA extract, the ITS locus was amplified using ITS1 and ITS4 primers (White et al. 1990). Specific *Cystobasidiomycetes* primers (Spribille et al. 2016) were not used because the purpose was also to detect all the fungal microbiome of our samples. However, this paper is only dedicated to *Cystobasidiomycetes*. The amplicons DNA library was prepared following the Ligation Sequencing Amplicons Native Barcoding Kit 96 V14 RevH (SQK-NBD114.96; ONT, https://store.nanoporetech.com/eu/productDetail/?id=native-barcoding-kit-96-v14). The manufacturer’s instructions were followed, with the following exceptions: the DNA Control Samples were not used and replaced by 1 µl of distilled water and a Carl Roth ATX 1.1 sample rotator mixer was used during the cleaning steps, instead of a Hula mixer. The amplicon library was loaded in a MinION MK1B R10.4.1 Flow Cell (ONT) and sequencing was launched with the MinKNOW software (ONT), for a maximum of three days. Sequenced reads were basecalled with the Guppy v. 6.5.7 toolkit (ONT; https://nanoporetech.com/software/other/guppy) for the first three experimental runs, or the Dorado v. 0.7.1 toolkit (ONT; https://github.com/nanoporetech/dorado/tree/release-v0.7) for the last experimental run.

### Bioinformatic analysis

The pipeline used to make ITS sequences was run via the Ghent University High Performance Computer (HPC, https://www.ugent.be/hpc/en), connected to the Vlaams Supercomputer Centrum (VSC, https://www.vscentrum.be/). For each barcode, reads were trimmed from their adapter and barcode sequence with Porechop (v. 0.2.4-intel-2019b-Python-3.7.4; Wick et al. 2017; https://github.com/rrwick/Porechop), before going through a quality-control, with the NanoComp software (v. 1.13.1-intel-2020b; De Coster and Rademakers 2023; https://github.com/wdecoster/nanocomp), where they were classified by quality and length. Reads were therefore filtered with the program NanoFilt (v. 2.6.0-intel-2020a-Python-3.8.2; De Coster et al. 2018; https://github.com/wdecoster/nanofilt) and only reads between 600 and 900 bp length and from Q15 or higher Phred score quality were retained. Finally, consensus sequences were made using NGSpeciesID (v. 0.3.0-foss-2023a; Sahlin et al. 2021; https://github.com/ksahlin/NGSpeciesID); Primers sequences were trimmed at this stage, sequences were polished with the Medaka tool (ONT) and an abundance ratio of 0.1% was set (i.e. reads clusters larger than 0.1% of the total reads fraction were kept). Consensus sequences supported by less than 10 reads were discarded.

The remaining consensus sequences were subjected to MegaBlast search (Madden 2002) against the Core Nucleotide database in GenBank and *Cystobasidiomycetes* sequences were retrieved and deposited in GenBank (Suppl. material 4: table S2). Other fungal sequences were kept aside for future studies (Borgato et al. Unpublished).

An exhaustive search was carried out (in November 2025) using the ‘Taxonomy’ tool in GenBank, retrieving all ITS sequences identified as *Cystobasidiomycetes* (including sequences from type materials, when available; Suppl. material 4: table S3). In addition, for each *Cystobasidiomycetes* sequence, the first 100 MegaBlast hits were retained to add unnamed *Cystobasidiomycetes* sequences (i.e. sequences named as “Uncultured fungus”, “Fungal sp”, etc.). Sequences that were identified as *Cystobasidiomycetes*, but revealed to be other fungi, were removed from the dataset and are listed in Suppl. material 4: tables S4a, b; *Cystobasidiomycetes* sequences that were erroneously assigned to other taxa are listed in Suppl. material 4: table S4c. The dataset included 2771 sequences, which were all classified by their reported source of origin (‘Environment’, ‘Animal’, ‘Plant’, ‘Fungus’ or ‘Lichen’), based on information found in the literature, or on public databases, such as GenBank (https://www.ncbi.nlm.nih.gov/genbank/) the Westerdijk Institute (CBS; https://wi.knaw.nl/) or the Japan Collection of Microorganisms (JCM; https://jcm.brc.riken.jp/en/).

*Cystobasidiomycetes* sequences detected from lichens were essentially produced by Spribille et al. (2016), Černajová et Škaloud (2019), Mark et al. (2020), Tagirdzhanova et al. (2021), Nguyen et al. (2023), Pérez et al. (2024), Millanes et al. (2025) and Stoppiello et al. (2025), while *Cystobasidiomycetes* sequences detected from other sources were essentially published by Hamamoto et al. (2002), Inácio et al. (2002), Scorzetti et al. (2002), Mahdi (2006), Velmurugan et al. (2013), Leite et al. (2014), Vu et al. (2016), Tsuji et al. (2017), Khunnamwong et al. (2018), Parra (2019), Parra and Aime (2019), Li et al. (2020), Akan et al. (2020), Gao et al. (2020), Haelewaters et al. (2020), Marčiulynas et al. (2020), Sun et al. (2020), Meng et al. (2022), Molnár et al. (2022), Kobayashi et al. (2024) and Zhang (2025). The reference list of all studies which produced *Cystobasidiomycetes* ITS sequences can be found in Suppl. materials 1–4, at the bottom of Suppl. material 4: table S3.

**Figure 2.**
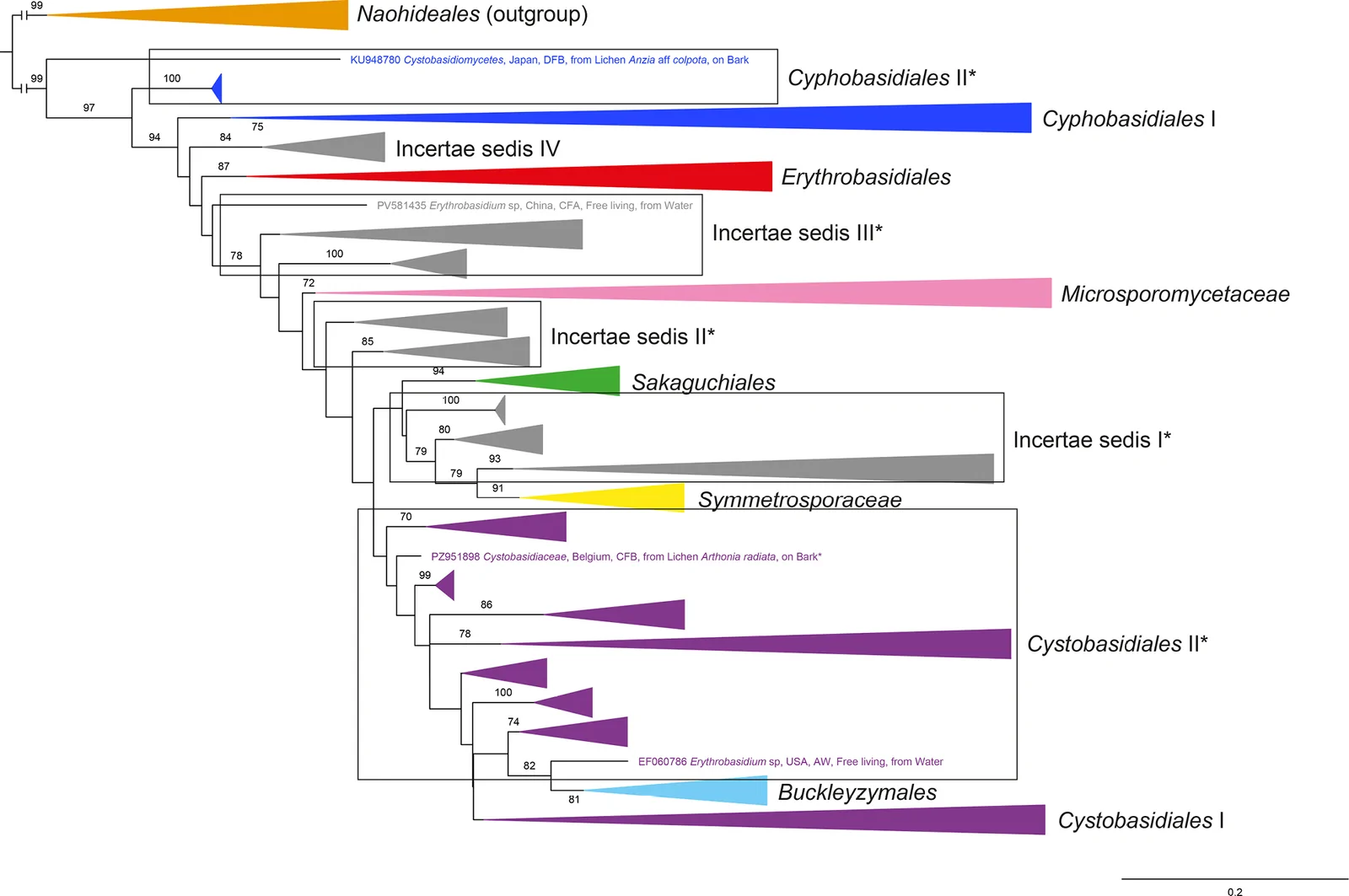
Overview of the majority-rule consensus tree of *Cystobasidiomycetes* from ML ultra-fast bootstrap analysis based on ITS data set. The clades (coloured) represent *Cystobasidiomycetes* orders. Only bootstrap values > 70% are shown. The complete and detailed phylogenetic tree is available at Suppl. material 1. Groups marked with an asterisk (*) are paraphyletic. “Incertae sedis” groups (in grey) represent *Cystobasidiomycetes* of uncertain classification.

**Figure 3.**
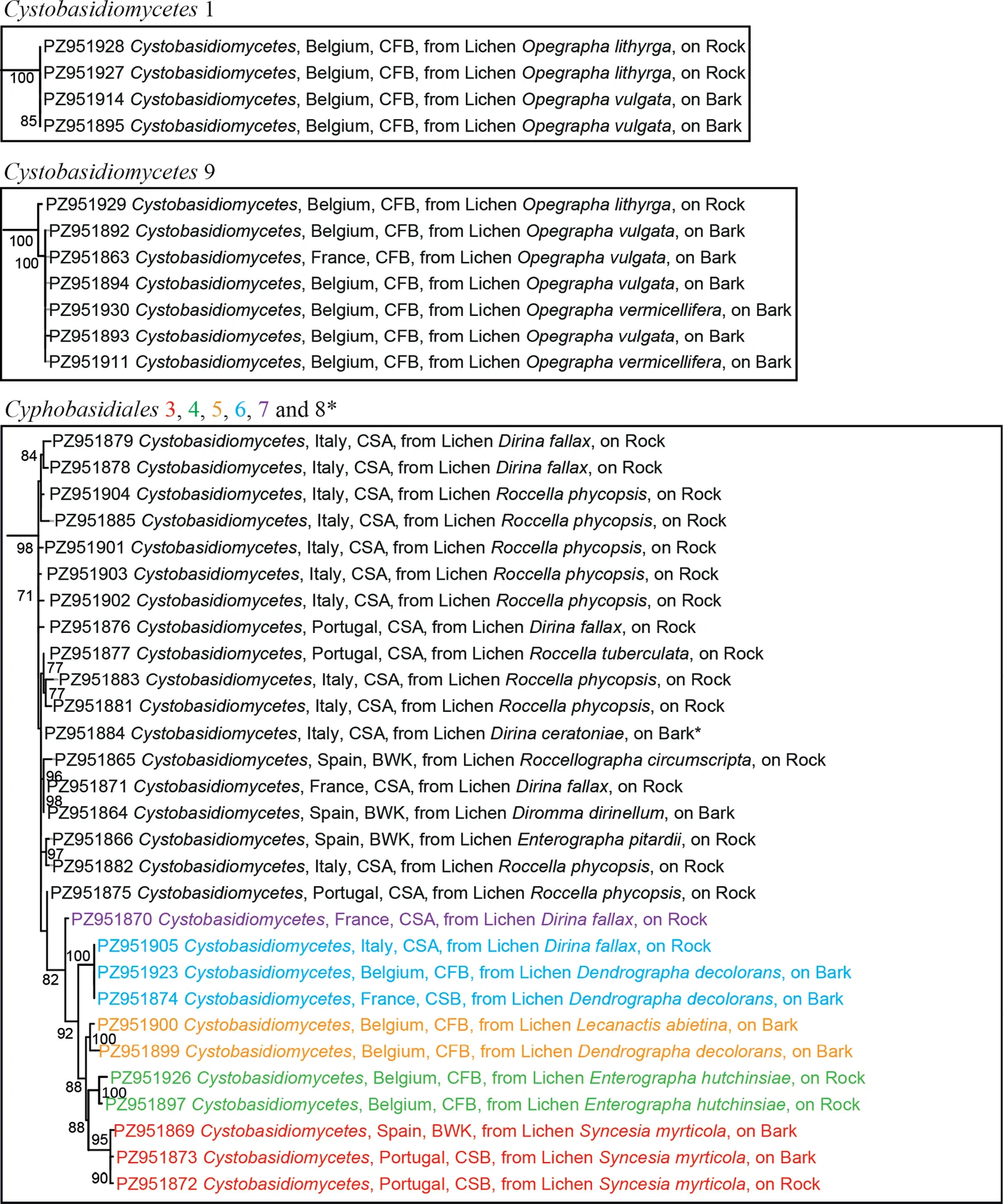
Three new *Cystobasidiomycetes* lineages formed by sequences from this study. The OTUs’ names are indicated above the lineages.

Sequences were automatically aligned using MAFFT v7.305 (Katoh 2002) on the CIPRES Web Portal (Miller et al. 2011), and manually improved using Mesquite 3.61 (Maddison and Maddison 2015) and PhyDe v. 0.9971 (Müller et al. 2010). Terminal ends of sequences were excluded from the dataset and ambiguous regions were excluded. Phylogenetic analysis was carried out using maximum likelihood (ML) inference, to build a worldwide *Cystobasidiomycetes* phylogenetic tree (Figs 2, 3, Suppl. material 1). The clade *Naohideales* was used as the outgroup, as done by Schoutteten et al. (2024), and consisted of six sequences of *Naohidea* spp. (DQ911616, EU870091, MK536639, NR_121324, OK039352 and OP954744; Matheny et al. 2006; Akulov et al. 2022; Canini et al. 2020; Zachow et al. 2009; Hui and Chai Unpublished). The program IQ-TREE 3 (Wong et al. 2025) was used on the IQ-TREE CIBIV (Center for Integrative Bioinformatics of Vienna, Austria) web server (Trifinopoulos et al. 2016), under the GTR-GAMMA model. Node supports of the maximum likelihood analyses were estimated by 1000 ultra-fast bootstrap replications (Hoang et al. 2018). Tree image was obtained with FigTree v. 1.4.2 (Rambaut 2012), annotated with ITOL v. 7 tool (Letunic and Bork 2024), and edited with Adobe Illustrator v. 25.41 (Adobe Inc 2019).

### OTUs delimitation, bipartite diagrams, rarefaction curves and statistical analyses

The number of OTUs was delimited by using the program Mothur (v. 1.48.1; Schloss et al. 2009), based on sequences similarity, with three thresholds: 97%, 98% and 99% of similarity. Taxonomically and phylogenetically curated OTUs were delimited by comparing the three thresholds, in combination with the phylogenetic tree topology, with the aim of maximizing monophyly. Sequences from type specimens were also used as references to assist in delimiting known species.

**Figure 4.**
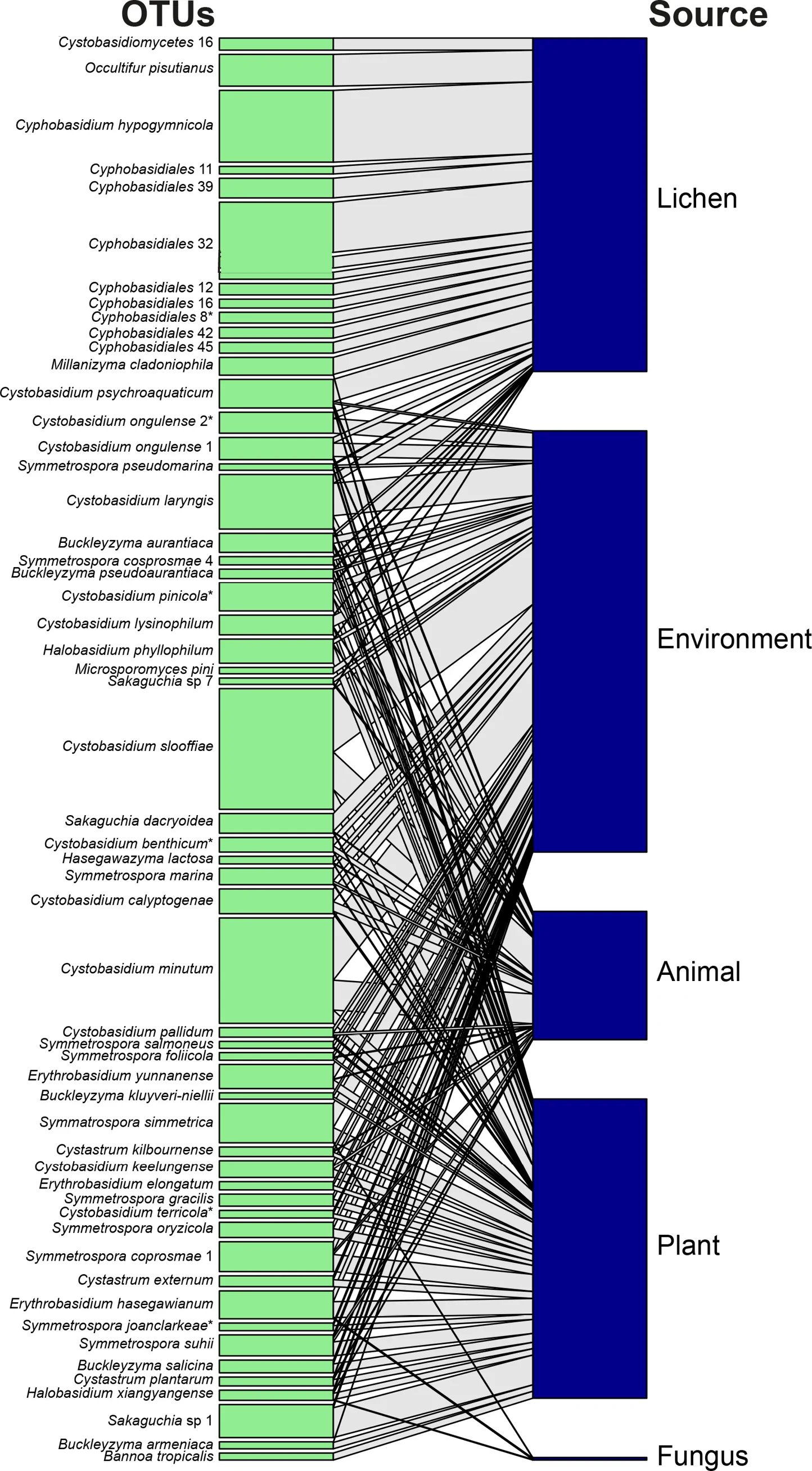
Bipartite network showing *Cystobasidiomycetes* OTUs (green; only OTUs represented by more than 10 sequences are shown) and their source of origin (blue). OTUs names are the ones from Suppl. material 4: tables S2-S3 and they are numbered from bottom to top of the phylogenetic tree (Suppl. materials 1, 2). The width of the links is proportional to the number of specimens forming the association. OTUs with an asterisk (*) are paraphyletic.

**Figure 5.**
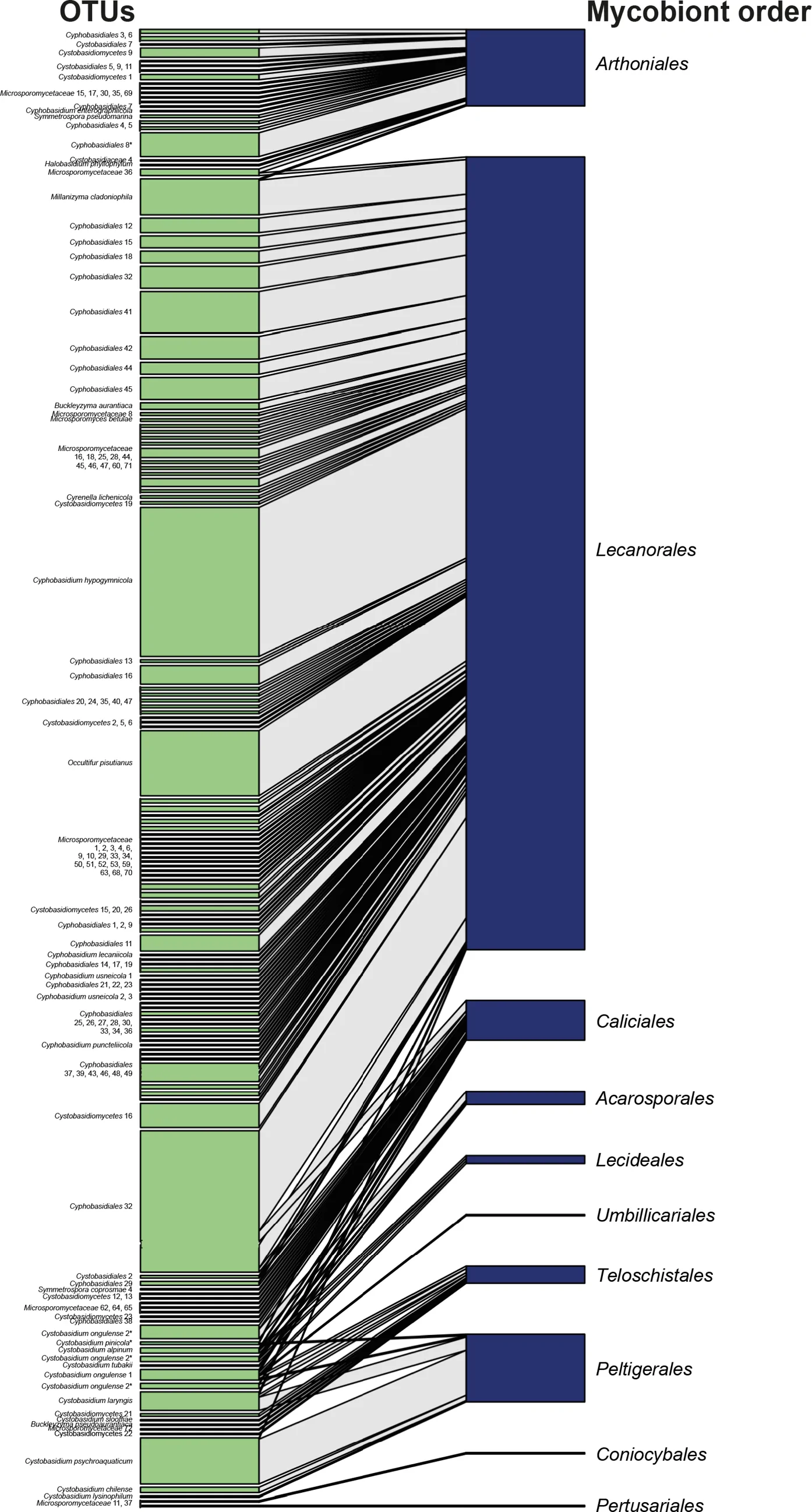
Interaction network between *Cystobasidiomycetes* OTUs (green) and mycobionts (blue) at order level. OTUs names are the ones from Suppl. materials 1, 2 and they are numbered from bottom to top of the phylogenetic tree (figs S1-S2). The width of the links is proportional to the number of specimens forming the association. OTUs with an asterisk (*) are paraphyletic.

**Figure 6.**
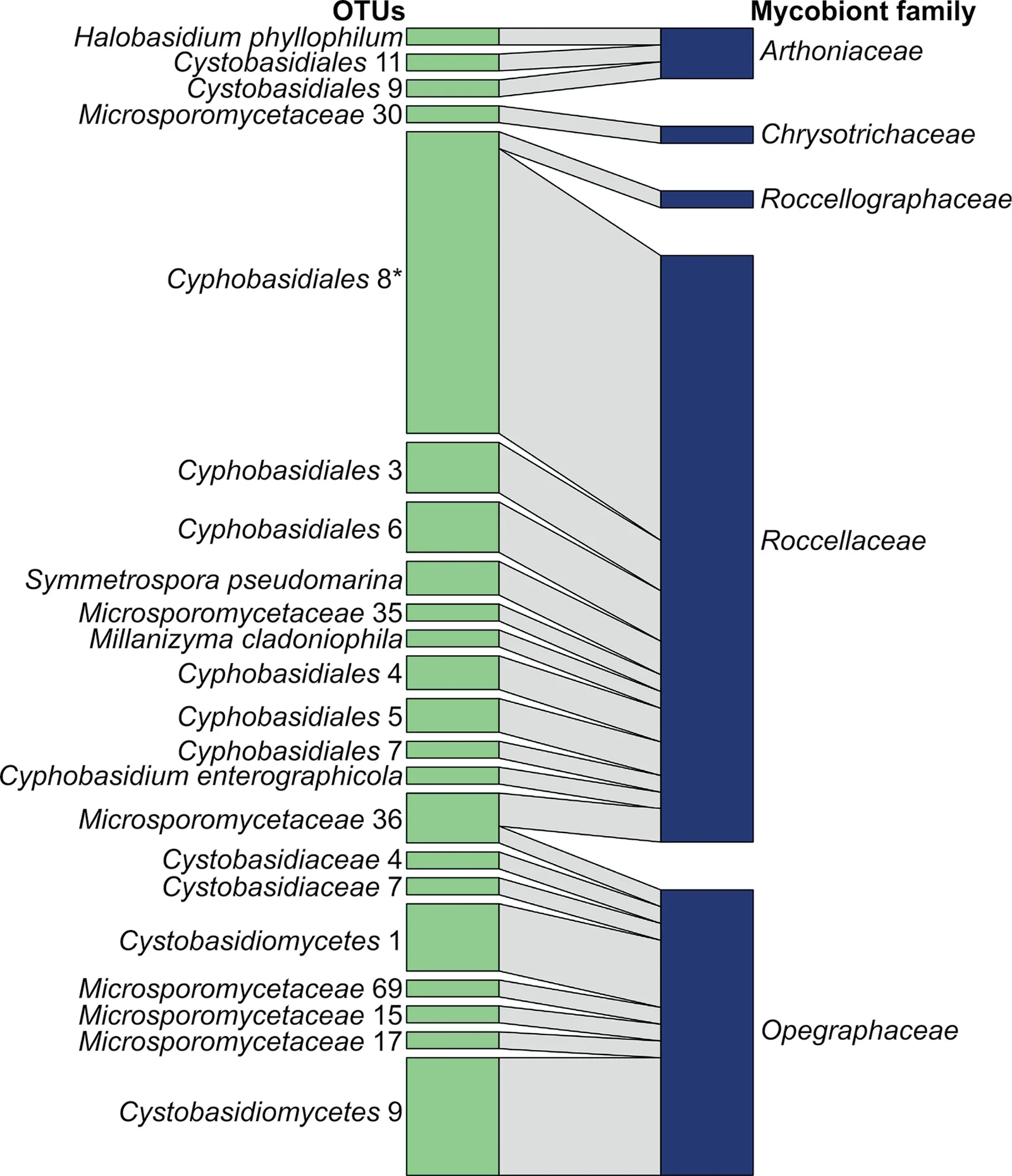
Interaction network between *Cystobasidiomycetes* OTUs (green) and *Arthoniales* mycobionts (blue) at family level. OTUs names are the ones from Suppl. materials 1, 2 and they are numbered from bottom to top of the phylogenetic tree (figs S1-S2). The width of the links is proportional to the number of specimens forming the association. OTUs with an asterisk (*) are paraphyletic.

**Figure 7.**
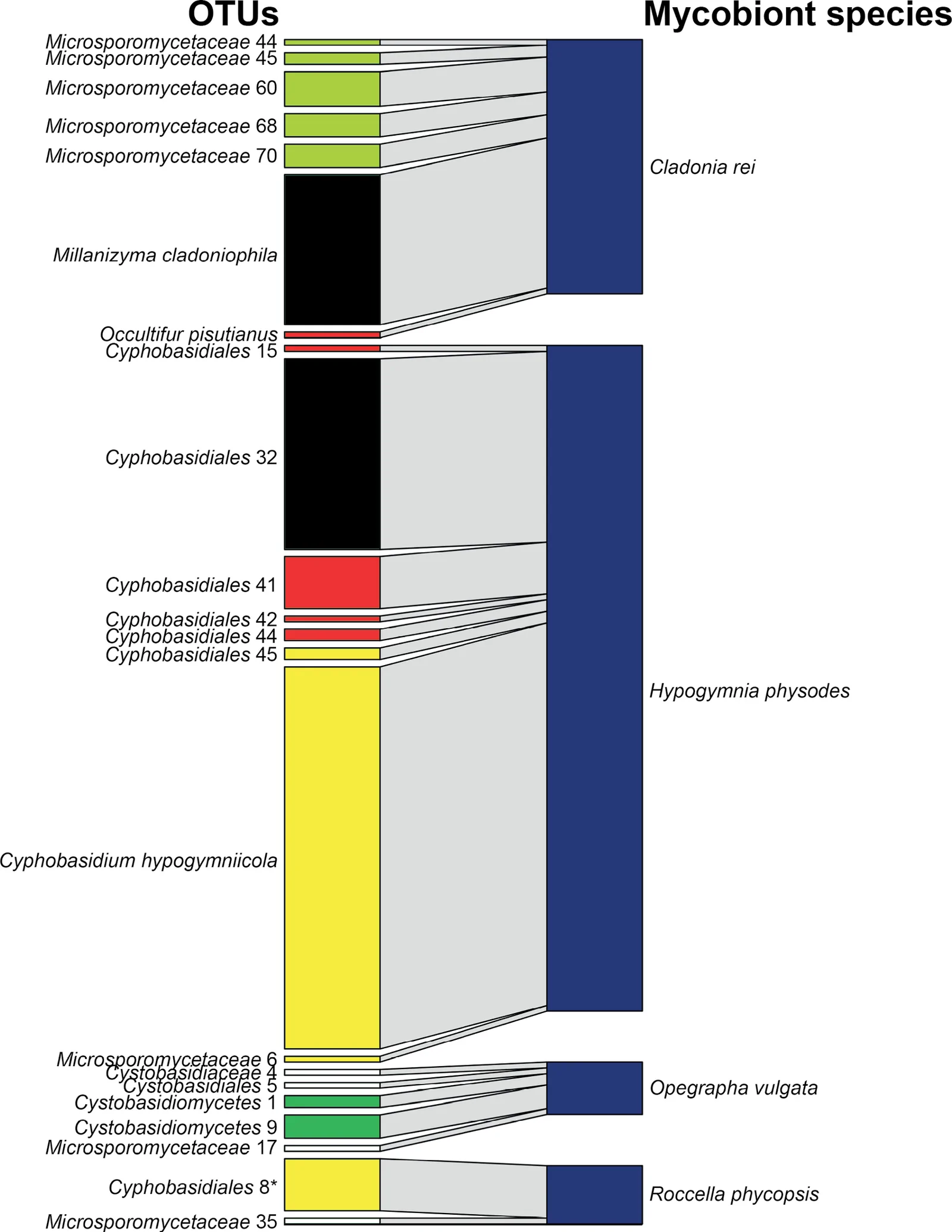
Interaction network showing a selection of four mycobiont species (right, blue) and their *Cystobasidiomycetes* OTUs (left). OTUs names are the ones from Suppl. materials 1, 2 and they are numbered from bottom to top of the phylogenetic tree (figs S1-S2). The width of the links is proportional to the number of specimens of the four mycobionts forming the association. OTUs with an asterisk (*) are paraphyletic. OTUs are coloured according to their degree of mycobiont selectivity (Black: not selective; yellow: order-selective; red: family-selective; dark green: genus-selective; light-green: species-selective; white: singleton).

The OTUs were used to build bipartite network graphs, with the Bipartite Network Analysis program (Greenville 2018). A first bipartite network was constructed by plotting the *Cystobasidiomycetes* OTUs against their source of origin (environment, animal, plant, fungus or lichen) to assess the proportion of OTUs exhibiting unique versus multiple sources (Fig. 4). Additional bipartite networks focused on lichen-associated fungi were used to visualize the associations between mycobionts and *Cystobasidiomycetes*, and to evaluate their degree of selectivity in the mycobiont choice and vice-versa (Figs 5, 6, 7).

For each tree clade (i.e. *Cystobasidiomycetes* orders) including sequences from lichen-associated *Cystobasidiomycetes*, rarefaction analyses were performed on mycobiont abundance (counts) as a function of the number of samples per clade, using iNEXT online (Chao et al. 2016; Hsieh et al. 2016), to determine whether the sampling effort was enough to estimate selectivity in clades (Suppl. material 3). A non-asymptotic approach based on interpolation and extrapolation was applied with default options and 70 bootstrap replications to calculate 95% confidence intervals to obtain sampling-coverage curves. Rarefaction curves were also made on *Arthoniales*-associated *Cystobasidiomycetes* sequences from this study (Suppl. material 3).

**Figure 8.**
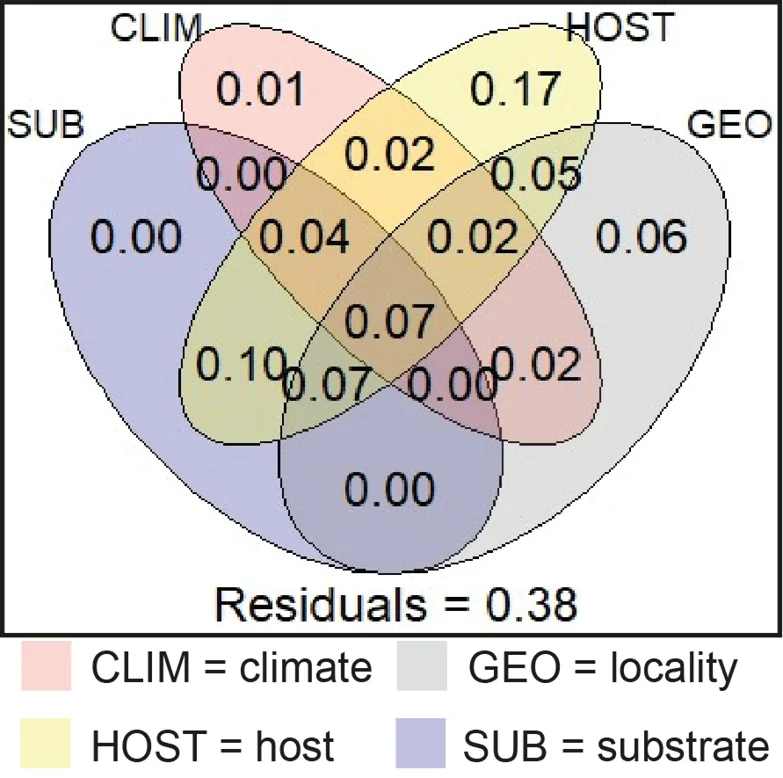
Venn’s diagram showing variation partitioning results on *Cystobasidiomycetes* full dataset.

**Figure 9.**
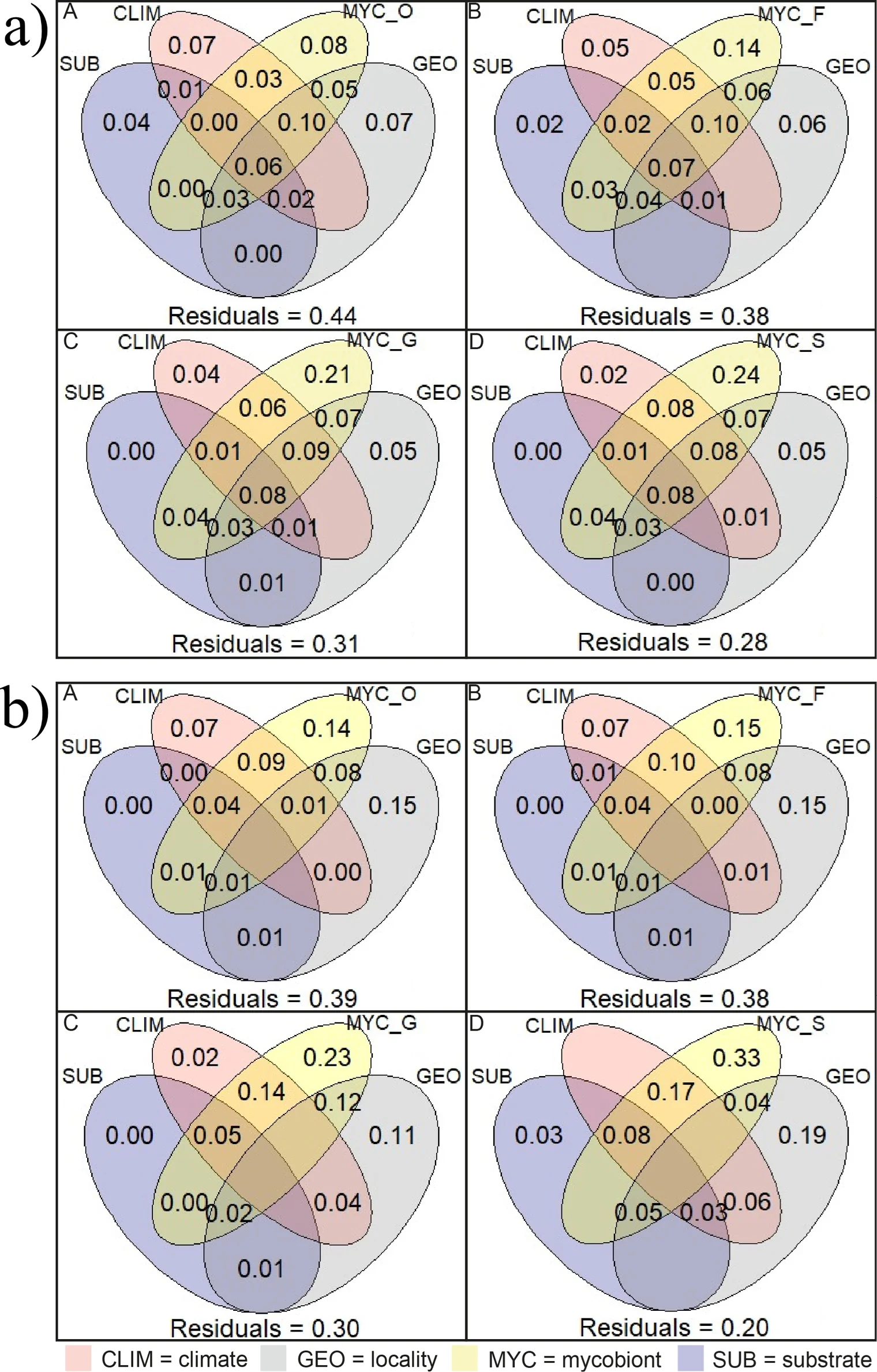
Venn’s diagrams showing variation partitioning results on lichen-associated *Cystobasidiomycetes* (**a**) and *Cyphobasidiales* (**b**) datasets, at mycobiont order (**A**), family (**B**), genus (**C**), and species (**D**) level.

To assess the relative contributions of substratum, climate, *Cystobasidiomycetes* host and geographic location to patterns in the *Cystobasidiomycetes* phylogeny, for all sequences, their geographic location, their host (where relevant), their substratum (or host substratum) and their climatic zone were registered, when available. When precise geographic coordinates were not available, an estimation was made, based on the information available in the literature, on GenBank, or on databases. For climate, the Köppen-Geiger classification (Beck et al. 2018) has been used, and sequences were assigned to the corresponding climatic zone thanks to their locations, where possible. A variation partitioning (Fig. 8) was performed in redundancy analysis (RDA) in R v. 4.4.2 (https://www.r-project.org/), using the *varpart* function in vegan R package version 2.5.7 (Oksanen et al. 2020). The phylogenetic distance of the dataset was calculated and a distance matrix was made using the online program PATRISTIC (Fourment and Gibbs 2006). The latter was transformed into principal coordinate analysis (PCoA) components using the *pcoa* function in ape R package version 5.5 (Paradis 2012; Paradis and Schliep 2018). A total of 1098 PCoA axes that showed positive eigenvalues were used for the variation partitioning analysis. Principal component axes (PCA) were calculated using the *rda* function in Vegan, separately on substratum, climate, and *Cystobasidiomycetes* host (Environment, Animal, Plant, fungus and lichen), coded as presence/absence dummy variables. The number of resulting PCA per variable is included in Suppl. material 4: table S5. The geographic distance matrices were transformed into principal coordinates of neighbourhood matrix (PCNM) components using the *pcnm* function in Vegan (Borcard and Legendre 2002; Borcard et al. 2011). The 165 PCNM components showing positive eigenvalues were kept for the variation partitioning analysis. Samples with missing ecological, geographic or mycobiont data were excluded from the analyses, resulting in a dataset of 2002 samples. The same statistical analysis was repeated on the same phylogeny, but selecting two smaller datasets, including only lichen-associated *Cystobasidiomycetes* (746 samples) and *Cyphobasidiales* (443 samples) respectively, to identify the relative contributions of substratum, climate, mycobiont host (at order, family, genus and species levels) and geographic location (Fig. 9). Sequences for which information on geographic location, substratum, climate or host were not available, were excluded from the analysis.

## Results

### *Cystobasidiomycetes* worldwide phylogeny and OTUs delimitation

The final alignment consisted of 858 patterns, with positions 1, 20–88, 559–579, 629–644, 732–740 and 788–829 considered as ambiguously aligned, which resulted in 158 excluded positions and 700 included positions. The derived *Cystobasidiomycetes* ITS worldwide phylogenetic tree (Fig. 2, Suppl. material 1) is based on 79 sequences from this study and 2692 selected from GenBank, for a total of 2771 sequences (including outgroup), distributed among the eight *Cystobasidiomycetes* orders and four groups of uncertain classifications (Fig. 2, Suppl. material 1). All orders in the phylogeny are recovered as monophyletic, with the exceptions of *Cyphobasidiales* and *Cystobasidiales*, which appear paraphyletic and each split into two distinct groups. Within *Cyphobasidiales*, most taxa form a monophyletic group; however, two OTUs (“*Cyphobasidiales* 48” and “*Cyphobasidiales* 49”) cluster separately, constituting the group “*Cyphobasidiales* II*”. *Cystobasidiales* largely form a monophyletic group, although *Buckleyzymales* are nested within them; but this relationship is unsupported. The observed paraphyly is likely an artefact of the phylogeny based solely on the ITS region. In contrast, the revision of *Cystobasidiomycetes* by Schoutteten et al. (2024), based on seven loci, supports the monophyly of both *Buckleyzymales* and *Cystobasidiales*. In addition, while *Cyphobasidiales* are closely related to *Erythrobasidiales*, the separation between these two orders is not clear, and their classification remains somewhat uncertain (Kachalkin et al. 2019; Schoutteten et al. 2024). However, for the purposes of this research, the two orders will be treated as distinct.

For this complete dataset, species delimitation with Mothur yielded 294 OTUs at the 97% threshold, 421 OTUs at the 98% threshold and 540 OTUs at the 99% threshold (Suppl. material 2). The number of conflicts was not high: in 67 cases, OTUs generated with 97% threshold were different from the ones generated with the 98% threshold, which were different from the ones generated with the 99% threshold in 49 cases; 84 differences were registered between the 97% and 99% thresholds (Suppl. material 2). In these cases, the following criteria were applied when making taxonomically and phylogenetically curated OTUs: when GenBank sequences had a species name and formed coherent groups (especially in presence of a type sequence), the OTU was easily adapted according to the species names (as, for example, with *Cystobasidium alpinum*, *Cystobasidium oligophagum*, *Occultifur pisutianus* or *Symmetrospora rhododendri*; Suppl. material 2), despite the risk of misidentified sequences. In case of unidentified sequences at species level, a conservative approach was used, giving priority to the less diverse OTU delimitation, to avoid potential artificial species and an overestimation of the true diversity (Carstens et al. 2013); this is particularly important when working with sequences without specimens (lack of phenotype). In addition, consensus OTUs were established to maximise monophyly; however, this was not always possible due to the topology of the ITS phylogeny. In total, 376 consensus OTUs were defined, of which 137 included lichen-associated sequences. Among these, 23 were associated with *Arthoniales*, all derived from this study except for two (*Cyphobasidium enterographicola* from *Enterographa pallidella*, and OTU “*Cystobasidiales* 5”, from *Opegrapha vulgata*; Lendemer et al. 2019; Diederich et al. 2022b). Six of the consensus OTUs remained paraphyletic: “*Cyphobasidiales* 8*”, “Cystobasidium benthicum*”, “*Cystobasidium ongulense* 2*”, “Cystobasidium pinicola*”, “Cystobasidium terricola*” and “Symmetrospora joanclarkeae*” (Suppl. material 2).

### *Cystobasidiomycetes* occurrence in collected samples

A total of 79 *Cystobasidiomycetes* sequences were detected in 60 samples from this study (ca. 35% all the samples; Suppl. material 4: table S1, S2), including 47 samples belonging to *Arthoniales* and 13 to non-*Arthoniales* lichens. They were assigned to 39 OTUs (Suppl. material 4: table S2). Of these, 21 OTUs were found in *Arthoniales* samples and 18 in other lichens.

*Cystobasidiomycetes* were unevenly distributed across the dataset. Lichen specimens, contained between one and three OTUs and yielded between 10 and 105.854 reads (Suppl. material 4: table S2). Two specimens (LB325 *Xanthoria parietina* and PD19760 *Usnea esperantiana*) harboured three OTUs each, while seven specimens (DE27495 *Phaeophyscia orbicularis*, DE27454 *Hypogymnia tubulosa*, LB48 *Opegrapha vulgata*, DE15963 *Fouragea filicina*, LB46 *Enterographa hutchinsiae*, DE23193 *Roccella phycopsis* and LB235 *Enterographa hutchinsiae*) contained two OTUs each. All remaining specimens contained a single OTU.

Reads abundance also varied markedly among samples. Specimen PD19760 (*Usnea esperantiana*) was the only sample with more than 100.000 *Cystobasidiomycetes* reads. Four specimens, LB217 (*Opegrapha vulgata*), DE23182 (*Dirina fallax*), LB325 (*Xanthoria parietina*) and DE27495 (*Phaeophyscia orbicularis*) contained between 3000 and 10000 reads. A further 13 specimens (PD19744 *Roccella maderensis*, LB48 *Opegrapha vulgata*, DE16273 *Syncesia myrticola*, DE23182 *Dirina fallax*, LB66 *Opegrapha vermicellifera*, LB250 *Arthonia radiata*, DE16857 *Syncesia myrticola*, DE22563 *Roccella phycopsis*, LB35 *Opegrapha vulgata*, LB235 *Enterographa hutchinsiae*, CGsn *Opegrapha vulgata*, LB258 *Dendrographa decolorans*, LB375 *Dendrographa decolorans*) yielded between 440 and 3000 reads, whereas the remaining 42 specimens contained fewer than 440 reads (Suppl. material 4: table S2).

Overall, these results indicate that *Cystobasidiomycetes* are neither evenly distributed nor ubiquitous across the sampled lichens, as they were not detected in most specimens and, when this was the case, showed high variability in both richness and read abundance.

### *Cystobasidiomycetes* global occurrence

The phylogenetic tree (Suppl. materials 1, 2) shows that 132, 37, 150, 11 and 137 OTUs include *Cystobasidiomycetes* sequences detected from the environment, animals, plants, fungi and lichens respectively, of which 69, 4, 89, 5 and 118 have a unique source. The other OTUs (91 in total) have either a mixed or an unknown origin (Suppl. material 2). The majority of *Cystobasidiomycetes* were collected on soil or bark (~81% of them; Suppl. material 4: tables S1–S3; for *Cystobasidiomycetes* associated with a host, including lichens, the substrate considered is the one of the hosts) and come from continental and oceanic climatic zones (more than 66% and 46% respectively).

*Cystobasidiomycetes* OTUs including lichen-derived sequences are found mainly in *Cyphobasidiales* and *Microsporomycetaceae* clades (54 and 46 OTUs respectively), 17 of them are from *Cystobasidiales*, a few are from *Buckleyzymales* and *Symmetrosporaceae* and an additional 16 OTUs come from *Cystobasidiomycetes* of uncertain classification. They are totally absent from *Erythrobasidiales* and *Sakaguchiales* clades. Among them, nearly 65% of the OTUs include *Cystobasidiomycetes* sequences coming from *Lecanorales*, followed by 17% associated with *Arthoniales* and 10% with *Caliciales*; the other orders are represented by less than 10% each. Around 48% and 43% of them include *Cystobasidiomycetes* sequences from fruticose and foliose lichens respectively, around 34% are from crustose ecorticate thalli; a small minority are from crustose corticate or fruticose ecorticate thalli (Suppl. material 4: tables S1–S3). The 23 OTUs from *Arthoniales* (21 from this study and 2 from GenBank; Lendemer et al. 2019; Diederich et al. 2022b) come principally from *Opegraphaceae* and *Roccellaceae*, followed by *Arthoniaceae* and one sequence from *Chrysotrichaceae* and *Roccellographaceae* each (Suppl. material 4: tables S2, S3). Eight of them, which concentrate the great majority of the obtained sequences, form three new lineages in the clades Incertae sedis I (OTUs “*Cystobasidiomycetes* 1” and “*Cystobasidiomycetes* 9”; four and seven sequences respectively) and *Cyphobasidiales* (OTUs “*Cyphobasidiales* 3, 4, 5, 6, 7 and 8*”; 29 sequences; Fig. 3); OTUs “*Cyphobasidiales* 3, 4, 5, 6, 7 and 8*” are largely represented by *Roccellaceae*-derived *Cyphobasidiales*, with only one exception (*Roccellographa circumscripta*, which is a *Roccellographaceae*) and are close to a sequence of *Cyphobasidium enterographicola* from an *Enterographa pallidella* (ON922982; Diederich et al. 2022b).

While the clade *Cyphobasidiales* is built of lineages with exclusively lichen-associated OTUs, *Buckleyzymales*, *Cystobasidiales*, *Microsporomycetaceae* and *Symmetrosporaceae* contain lineages with OTUs from several sources (either one or mixed origins). Lichen-derived *Cystobasidiomycetes* belong, in most of the cases, to strict endolichenic OTUs, with only 19 mixed-sourced OTUs out of 137 (a much lower proportion than OTUs from other origins; see above). These few cases (Fig. 4, Suppl. materials 1, 2), include, for example, one sequence of *Millanizyma cladoniophila* (an essentially lichen-associated OTU) associated with *Picea abies* and one sequence of “*Microsporomycetaceae* 68” associated with an *Orchidaceae* plant. Vice-versa, some *Cystobasidiomycetes* sequences coming from lichens are also found in OTUs of essentially other sources, like, for example, in a sequence of *Halobasidium phyllophilum* associated with *Arthonia atra*, one sequence of *Buckleyzyma pseudoaurantiaca* associated with *Xanthoria parietina*, two sequences of *Symmetrospora pseudomarina* associated with *Roccella maderensis* or one sequence of “*Microsporomycetaceae* 30” associated with *Chrysothrix candelaris*. These exceptions likely represent airborne dispersal cells that were accidentally deposited on the lichen thallus. Finally, several additional OTUs include *Cystobasidiomycetes* sequences of mixed origin, such as “*Cystobasidium ongulense* 1 and 2*”, *Cystobasidium alpinum*, *Cystobasidium laryngis*, *Cystobasidium psychroaquaticum* and *Buckleyzyma aurantiaca*. The latter represent probably generalist OTUs, with or without lichen-derived sequences.

### *Cystobasidiomycetes* and mycobionts selectivity

The degree of selectivity of *Cystobasidiomycetes* in the mycobiont choice, and vice-versa, is variable and several patterns are observed. While some *Cystobasidiomycetes* OTUs are not selective at all and are associated with mycobionts from several orders (like OTUs “*Cystobasidium ongulense* 1 and 2*”, “*Cyphobasidiales* 29 and 32”, “*Microsporomycetaceae* 36”, etc.; Figs 5, 6, 7, Suppl. materials 1, 2), others are selective either at mycobiont order (like OTUs *Cyphobasidium hypogymniicola* or *Occultifur pisutianus*, associated with 113 and 50 sequences of *Lecanorales* respectively; or OTU “*Cyphobasidiales* 8*”, associated with 18 sequences of *Arthoniales*), family (like OTUs “*Cyphobasidiales* 41/42/44”, associated with 31, 17 and 18 sequences of *Parmeliaceae* respectively), genus (like OTUs “*Cyphobasidiales* 16” and “*Microsporomycetaceae* 44”, associated with 14 sequences of *Bryoria* and seven sequences of *Cladonia* respectively) or species levels (like OTUs “*Cyphobasidiales* 3/11/12”, associated with three sequences of *Syncesia myrticola*, 12 sequences of *Evernia prunastri* and 11 sequences of *Alectoria sarmentosa*, or “*Microsporomycetaceae* 60/68/70”, associated with six, four and four sequences of *Cladonia rei* respectively).

In addition, other *Cystobasidiomycetes* OTUs are not completely selective, but have a preference for a certain mycobiont taxon, like OTUs *Cystobasidium psychroaquaticum* associated with 34 sequences of *Peltigera* and only one sequence of *Cladonia pocillum*, *Millanizyma cladoniophila* associated with 26 sequences of *Cladonia rei* and only one sequence of *Enterographa hutchinsiae*, *Cyphobasidium hypogymniicola* associated with several sequences of *Lecanorales* mycobionts, but the great majority of them being specimens of *Hypogymnia physodes* or *H. tubulosa*, “*Cyphobasidiales* 8*” associated with several sequences of *Arthoniales*, all *Roccellaceae*, except one of *Roccellographa circumscripta*, or “*Cyphobasidiales* 42” associated essentially with *Parmeliaceae*, but with a few sequences from *Lecanora* mycobionts (Figs 5, 6, Suppl. material 1, 2). Other lichenized fungi species seem also to be selective, but are represented by only one or very few sequences, for which the sampling should be improved for confirmation, as supported by rarefaction analysis (Suppl. material 3).

*Arthoniales*-associated OTUs are not associated with any other mycobiont order, with only two exceptions: *Millanizyma cladoniophila* associated with *Enterographa hutchinsiae* and *Cladonia rei*, and the recently new discovered OTU “*Microsporomycetaceae* 36”, associated with three *Arthoniales* (from this study) and two *Lecanorales* (Millanes et al. 2025). Six of them are selective to *Roccellaceae* (except one sequence from *Roccellographa circumscripta*), two of them are selective to *Opegrapha* (OTUs “*Cystobasidiomycetes* 1 and 9”; four and seven sequences respectively). The other 12 OTUs (only 12 sequences) are either singletons or share the same OTUs of non-endolichenic *Cystobasidiomycetes* (see explanations above).

A trend is also observed in mycobionts, as lichens are also not randomly associated with *Cystobasidiomycetes* OTUs. Mycobionts like *Alectoria sarmentosa*, *Bryoria* spp. or *Evernia prunastri* are only associated with *Cystobasidiomycetes* OTUs of the order *Cyphobasidiales*. Others, like *Hypogymnia* spp. are associated with several *Cystobasidiomycetes* orders, but the great majority of the specimens are linked to *Cyphobasidiales*. On the other side, other mycobionts species from other orders (except *Arthoniales* and *Caliciales*) are never associated with *Cyphobasidiales* partners, as it is the case for *Peltigera* spp. *Acarosporales* or *Teloschistales*, exclusively associated with *Cystobasidiales*. Even among *Lecanorales*, *Cladonia* spp. (except two sequences), are nearly all associated with *Microsporomycetaceae* and one lineage of uncertain classification (Figs 5, 7, Suppl. materials 1, 2). A similar pattern is also visible in *Arthoniales* mycobionts, as *Dirina* spp. and nearly all *Roccella* spp. are associated with *Cyphobasidiales* (OTU “*Cyphobasidiales* 8*”), while *Arthoniaceae* and *Opegraphaceae* are associated with *Cystobasidiomycetes* from other lineages (Figs 6, 7, Suppl. materials 1, 2), separated from each other. Inside the *Cyphobasidiales* order a preference of mycobionts for certain OTUs is also observed, as, for example, *Hypogymnia* spp. are mostly concentrated in OTUs *Cyphobasidiales* 32 and *Cyphobasidium hypogymniicola*, *Alectoria sarmentosa* and *Bryoria* spp. are associated with OTUs *Cyphobasidiales* 12–17, which cluster together in a supported lineage (100% of bootstrap).

Rarefaction and sampling coverage curves (Suppl. material 3) clearly show that the sampling is insufficient in *Arthoniales*, as well as in other lichenized fungal orders. Even if the sampling coverage curve reaches a plateau for some of the clades, the latter is lower than 100%, which means that there are still a lot of new lichen-associated *Cystobasidiomycetes* to discover.

### Variation partitioning results

The variation partitioning on the entire dataset (Fig. 8) shows that the distribution of *Cystobasidiomycetes* is principally explained by their host (17%) and a combined effect of the latter and their (host) substrate (10%). Other factors also explain the variance, but all at low percentages. Geographical location contributes to 6%, while the effects of substrate and climate alone are practically negligible (0% and 1% respectively). Combined effects of substrate, climate and host (and geography; 4% and 7% respectively), of host and climate (and geography; 2% each) and of host and geography (5% respectively) explain also each a small part of the variance. A little more than 1/3 of the variance (38%) remains unexplained.

The variation partitioning analyses on lichen-associated *Cystobasidiomycetes* and *Cyphobasidiales* datasets (Fig. 9) show also a major contribution of mycobiont host, which is higher at mycobiont species level (24% and 33% respectively). While the geographic location has a similar effect compared to the entire dataset with the lichen-associated *Cystobasidiomycetes* dataset (5–7%), the latter rises to 11–19% with the *Cyphobasidiales* dataset. In addition, a significant combined effect of climate and mycobiont taxonomy is observed on the *Cyphobasidiales* dataset (from 9% at mycobiont order level to 17% at mycobiont species level), which is weaker when including all lichen-associated *Cystobasidiomycetes* (3–8%). A significant combined effect of mycobiont taxonomy, climate and geography is also observed on the lichen-associated *Cystobasidiomycetes* dataset (from 10% at mycobiont order level to 8% at mycobiont species level). Some of the other factors explain also small part of the variance, but all under 10%, others do not contribute to the variance (0%; Fig. 9). Residuals on the lichen-associated *Cystobasidiomycetes* and *Cyphobasidiales* datasets are from 44% and 39% at mycobiont order level, to 28% and 20% at mycobiont species level respectively, meaning the variance is significantly better explained with these two datasets than the entire dataset.

### Thallus type and cortex presence

**Figure 10.**
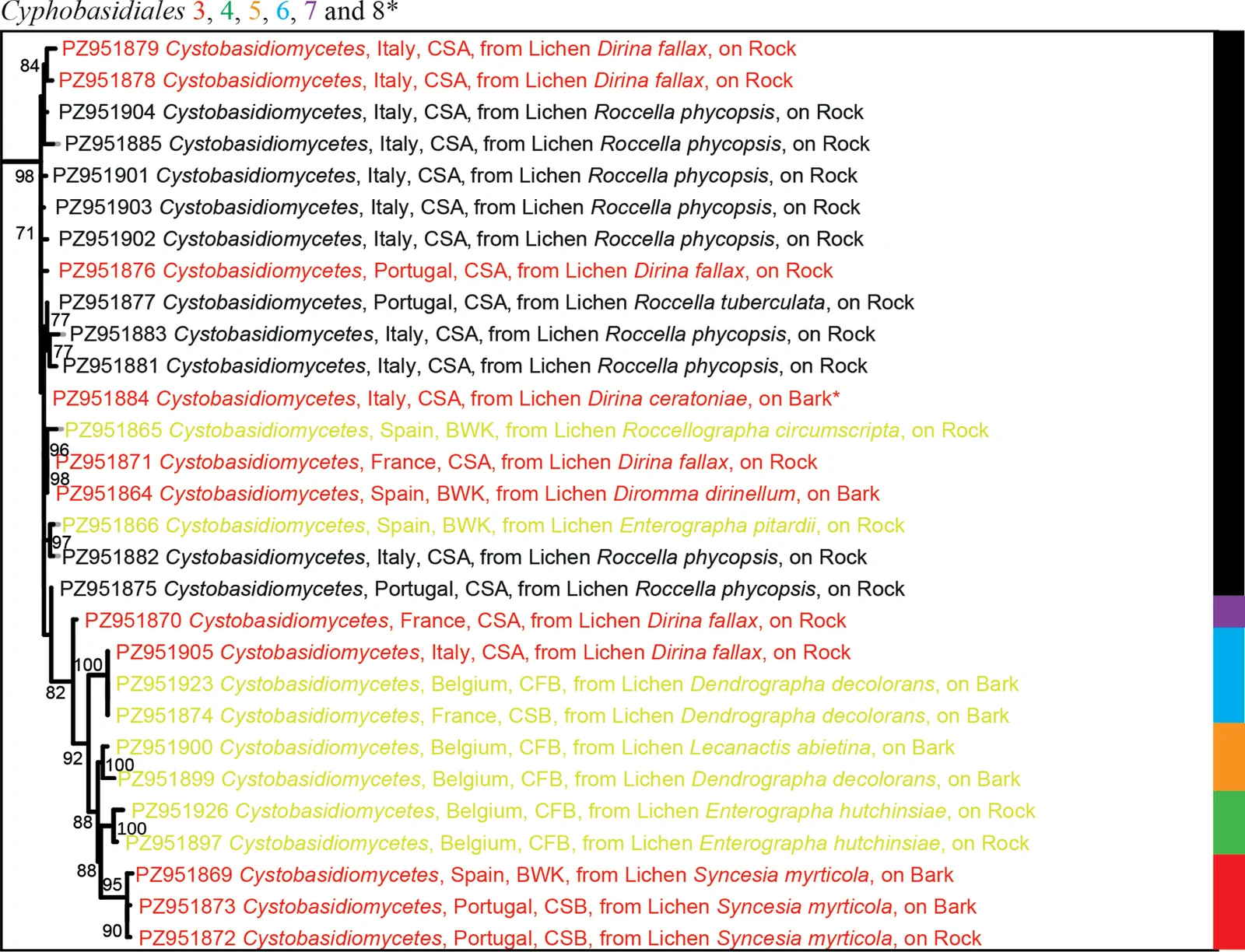
Mixture of fruticose (black) and crustose (corticate: red; ecorticate: yellow) mycobiont partners in one of the three new lineages, in *Cyphobasidiales* I clade. Coloured lines on the right represent the OTUs.

Mycobiont thallus type and cortex presence/absence do not seem to influence *Cystobasidiomycetes*’ mycobiont choice. Phylogenetic tree and OTUs composition (Fig. 10, Suppl. materials 1, 2, Suppl. material 4: tables S1–S3) clearly show that *Cystobasidiomycetes* OTUs can be associated with lichenized fungi of several thallus type and both corticate and ecorticate thalli, which is also the case of *Arthoniales*-derived OTUs, as shown in OTUs “*Cyphobasidiales* 3–8*” (Fig. 10).

Some OTUs are, on the contrary, associated with only one lichen type, like OTUs “*Microsporomycetaceae* 60 and 68” (*Cladonia rei*: fruticose corticate; six and four sequences respectively), OTUs “*Cystobasidiomycetes* 1 and 9” (*Opegrapha*: crustose ecorticate; four and seven sequences), “*Cyphobasidiales* 3” (*Syncesia myrticola*: crustose corticate; three sequences) or “*Cyphobasidiales* 16” (*Bryoria*: fruticose corticate; 14 sequences), however, these cases are restricted to particular taxa. In addition, some of these OTUs still need more sampling, as demonstrated by rarefaction analysis (Suppl. material 3). Moreover, not all specimens of a lichen type from this study have *Cystobasidiomycetes*, as is the case with the species *Roccella phycopsis*: while all yeasts found in this species belong to OTU “*Cyphobasidiales* 8*” in clade *Cyphobasidiales* I (Figs 3, 10, Suppl. materials 1, 2, Suppl. material 4: tables S1-S2), not all specimens of *Roccella phycopsis* have them (Suppl. material 4: table S1).

## Discusssion

### *Cystobasidiomycetes* diversity and occurrence

The worldwide phylogeny and OTU analyses reveal a clear separation between *Cystobasidiomycetes* detected from lichens and those recovered from other sources (Fig. 4, Suppl. materials 1, 2). Our results provide three main insights into the diversity and ecology of lichen-associated *Cystobasidiomycetes*.

First, lichen-associated *Cystobasidiomycetes* from our samples are diverse but not ubiquitous. They were detected in only 35% of our specimens (Suppl. material 4: table S1) and with variable abundance (Suppl. material 4: table S2), indicating that they are neither evenly distributed nor ubiquitous. This finding is consistent with studies that also failed to detect these yeasts in all sampled lichens (e.g., Lendemer et al. 2019; Cometto et al. 2022, 2024; Nguyen et al. 2023), but contrasts with studies reporting a more widespread occurrence (e.g., Spribille et al. 2016; Černajová and Škaloud 2019). At the same time, our data substantially expand the known diversity of *Arthoniales*-associated *Cystobasidiomycetes*. We recovered 21 additional OTUs containing *Arthoniales*-derived sequences, eight of which belong to three new lineages (Figs 3, Suppl. material 1). With only two exceptions, all OTUs recovered from *Arthoniales* are distinct from previously reported OTUs (Suppl. materials 1, 2; Černajová and Škaloud 2019; Millanes et al. 2025). Given that only two *Cystobasidiomycetes* sequences from *Arthoniales* were previously known (Lendemer et al. 2019; Diederich et al. 2022b), these results suggest that the diversity of *Cystobasidiomycetes* associated with this lichen order remains largely unexplored.

Second, mixed OTUs indicate that ecological associations are not always exclusive. In 19 cases, lichen-derived sequences belonged to OTUs containing sequences from other sources (see Results). These OTUs may represent generalist taxa capable of occurring in different environments and hosts, but alternative explanations are also possible. OTUs dominated by lichen-derived sequences may be lichen-associated species, but include sequences detected outside lichens, whereas OTUs dominated by sequences from other sources may include environmental airborne *Cystobasidiomycetes* incidentally detected within lichens. The latter possibility is particularly relevant for corticolous crustose lichens, for which complete separation of the thallus from bark is often difficult, especially if the thallus is very thin, like the genus *Opegrapha*. In addition, since in our study we did not clean our thalli to avoid losing *Cystobasidiomycetes* accidentally, the probability to detect airborne spores of external *Cystobasidiomycetes* is higher. However, our worldwide phylogeny (Suppl. material 1) allows to determine with a reasonable degree of confidence which of the OTUs detected in our samples are truly lichen-associated or not. For example, the sequence of *Halobasidium phyllophilum* detected in a specimen of *Arthonia atra* (Suppl. material 4: table S2) clearly does not represent a fungus associated with the mycobiont, but rather one associated with the tree on which the lichen was growing (Suppl. material 1).

Lastly, ITS isolation source does not necessarily indicate ecological origin. More broadly, the source from which an ITS sequence was obtained cannot always be taken as a reliable information for the organism’s ecological lifestyle. This issue is particularly important for sequences originating from the environment (i.e. no host; Suppl. material 4: table S3): yeasts detected in air, dust, water, or soil may represent host-associated organisms transported into these environments. Moreover, animal-derived sequences may originate from the animal itself, its skin, or ingested material, while plant-derived sequences may originate from epiphytes, animal excrements, airborne cells, or soil communities deposited on plant surfaces. The “*Cystobasidium ongulense* 2” OTU illustrates this ambiguity, comprising sequences obtained from ice and from Antarctic lichens (Suppl. materials 1, 2, Suppl. material 4: table S3; Stoppiello et al. 2025). The greater intermixing of environment- and animal-derived OTUs compared with lichen-derived OTUs (Fig. 4) may therefore reflect both genuine generalism and uncertainty in assigning ecological origin. Plant-associated OTUs are less mixed, possibly because plants provide relatively stable substrates compared with mobile animals. Overall, ecological assignments based solely on environmental sequencing source should therefore be regarded as provisional, unless if supported by additional evidence from other reliable and verified sources.

### *Cystobasidiomycetes* and mycobiont selectivity

Lichen-associated *Cystobasidiomycetes* belong predominantly to *Cyphobasidiales*, but are also present in several other lineages, except in *Erythrobasidiales* and *Sakaguchiales*. Overall, their selectivity towards mycobionts appears limited. Only a minority of OTUs show apparent specificity at the mycobiont genus or species level (Spribille et al. 2016; Nguyen et al. 2023; Millanes et al. 2025; this study), consistent with the uneven distribution of *Cystobasidiomycetes* across study areas and mycobionts reported by Mark et al. (2020). Apparent specificity may also reflect insufficient sampling. For example, OTU “*Cystobasidiomycetes* 16”, initially reported only from *Cladonia* (Černajová and Škaloud 2019), was subsequently detected in other lichens (Mark et al. 2020; Cometto et al. 2022). Similarly, the newly identified lineages associated with *Opegrapha* may prove to be less specific as sampling increases. Rarefaction analyses indicate that sampling remains insufficient for most clades (Suppl. material 3).

Despite this generally low specificity, *Cystobasidiomycetes* do not appear to associate randomly with mycobionts, which is also supported by variation partitioning analysis, which detected an effect of mycobiont taxonomy on *Cystobasidiomycetes* distribution (Fig. 9b). For example, *Roccellaceae* are associated with *Cyphobasidiales*, whereas *Cyphobasidiales* were never detected in *Arthoniaceae* or *Opegrapha* spp. (Fig. 3, Suppl. material 1). Within *Lecanorales*, *Cyphobasidiales* are frequent in *Parmeliaceae* but rare in *Cladoniaceae*, including *Cladonia* spp., with only two exceptions (Spribille et al. 2016; Mark et al. 2020; Nguyen et al. 2023; this study). Even within *Cyphobasidiales*, *Alectoria*, *Bryoria*, *Evernia*, *Hypogymnia* and *Usnea* are mostly associated with separate OTUs. Thus, lichen-associated *Cystobasidiomycetes* appear neither strictly host-specific nor completely generalist, but rather show structured yet flexible partner choice, with individual OTUs potentially having preferred primary hosts and occasional secondary hosts.

Selectivity may also operate in the opposite direction. Our *Arthoniaceae* and *Opegraphaceae* samples were associated mainly with *Cystobasidiales*, *Microsporomycetaceae* and *Cystobasidiomycetes* of uncertain classification, whereas other *Arthoniales* families also hosted *Cyphobasidiales* (Fig. 3, Suppl. material 1). This suggests possible bidirectional selectivity, although broader *Arthoniales* sampling is needed to confirm this pattern.

This degree of selectivity is broadly comparable to that reported for some lichen-associated fungi, but less pronounced than in highly host-specific interactions. Lichenicolous heterobasidiomycetes are generally host-specific, whereas lichenicolous homobasidiomycetes may infect several lichen species while retaining preferences for particular host groups (Diederich et al. 2022b). Similarly, Łubek et al. (2019) found that lichenicolous fungi associated with *Parmeliaceae* differed from those occurring on *Cladoniaceae* and *Pertusariaceae*. Moreover, some *Tremella* and *Cystobasidiomycetes* species may be more host-selective in their lichenicolous, filamentous phase than in their asymptomatic yeast phase (Millanes et al. 2025).

Interestingly, photobionts also show structured partner choice. Trebouxioid photobionts associated with *Parmeliaceae* tend to be phylogenetically distinct from those associated with other mycobionts or preferentially associated with *Caliciaceae*, *Lecanoraceae* or *Physciaceae*, while trentepohlioid photobionts associated with *Arthoniaceae* are distinct from those of other *Arthoniales* (Mark et al. 2020; Borgato et al. 2022; Kosecka et al. 2022, 2026). *Cystobasidiomycetes* show similar patterns: OTUs associated with *Parmeliaceae* may also occur with *Lecanoraceae* or *Physciaceae* as secondary partners, as in *Cyphobasidium hypogymniicola* and “*Cyphobasidiales* 32” (Suppl. material 1, 2), while *Arthoniaceae*-derived yeasts are distinct from those associated with other *Arthoniales*. However, exceptions exist: *Cystobasidiomycetes* associated with *Enterographa hutchinsiae* do not cluster with those from *Opegrapha*, whereas their photobionts do (Borgato et al. 2022).

Overall, *Cystobasidiomycetes*, like photobionts and lichenicolous fungi, appear to exhibit structured but flexible partner choice, rather than strict host specificity or random associations. Our findings in our *Arthoniales* specimens confirm this trend. However, attention should be paid with our samples, since the thalli surfaces were not cleaned. The main issue could be detecting a lichen-associated *Cystobasidiomycetes* that is not truly associated with that mycobiont, but to a nearby mycobiont. Here again, our worldwide phylogenetic tree may help to detect such cases, like for example, a sequence of *Microsporomyces cladoniophilus* found in a specimen of *Enterographa hutchinsiae* (Suppl. material 1, Suppl. material 4: table S2), while all the other sequences forming that OTU come from *Cladonia rei* (Nguyen et al. 2023) and the nearby lichen-associated OTUs are linked to *Cladonia* specimens (Černajová and Škaloud 2019). Further sampling would also be useful.

### Effects of climate and substrate

The variation partitioning analysis on the total dataset revealed negligible contribution of climate and substrate, except for a minor effect when these factors were considered in combination with *Cystobasidiomycetes* host and/or geography (Fig. 8). The same analysis on lichen-derived *Cystobasidiomycetes* and *Cyphobasidiales* datasets allowed to unravel a combined effect of climate and mycobiont taxonomy, especially at mycobiont species level (Fig. 9). However, robust conclusions regarding these two factors could not be drawn based on the present study, because of an unbalanced dataset. *Cystobasidiomycetes* are predominantly represented by sequences originating from oceanic and continental climatic regions (zones C and D of the Köppen-Geiger classification; Beck et al. 2018), which do not differ markedly in their climatic conditions (Fig. 1). In contrast, fewer than one-third of the OTUs originate from other regions, and even fewer in the lichen-derived dataset (only 12 sequences come from a tropical environment; Suppl. material 4: table S3). Moreover, the dataset is unbalanced also for the substrate, with a predominance of OTUs (or hosts) from bark. OTUs from rock represent only 13% (Suppl. material 4: tables S1–S3) and only three sequences of lichen-derived *Cystobasidiomycetes* are from foliicolous lichens. In addition, half of the sequences (or hosts) from soil concern plant-derived *Cystobasidiomycetes*.

Therefore, the only hypotheses that could be proposed, based on the current dataset is that an effect of climate and substrate on *Cystobasidiomycetes* distribution is plausible, but underestimated. A similar issue is present in Mark et al. (2020) whose dataset is restricted to Estonia and Switzerland, regions with broadly comparable climatic conditions. A comparison with the dataset used by Borgato et al. (2022) to study trentepohlioid lichens shows that in the latter the number of algal sequences coming from tropical and extra-tropical regions is more or less balanced and the variation partitioning analysis carried out in that study showed that 35% of the variance is explained by a combined effect of climate and mycobiont species (fig. 5 in Borgato et al. 2022). In addition, a general trend is observed in *Cystobasidiomycetes* OTUs (Suppl. materials 1, 2), as lichen-derived *Cyphobasidiales* are predominantly associated with corticolous lichens, whereas *Cystobasidiales*, *Microsporomycetaceae* and lineages of uncertain classification are mainly associated with terricolous and saxicolous lichens, although corticolous lichens are also well represented within these groups. Therefore, additional saxicolous, terricolous, but especially foliicolous lichens, as well as lichens from tropical regions are absolutely needed to confirm these trends in the present study.

### No correlation with thallus architecture or cortex presence

This study did not reveal any clear correlation between the presence or absence of *Cystobasidiomycetes* and thallus morphology or the presence or absence of a cortex in *Arthoniales*. First, phylogenetic (Figs 10, Suppl. materials 1, 2) and network analyses (Fig. 6) indicate that different lichen-derived *Cystobasidiomycetes* OTUs are largely associated with mycobionts exhibiting a wide range of thallus morphologies, as reflected by the diversity of OTUs recovered (Suppl. materials 1, 2). Within *Arthoniales*, OTUs “*Cyphobasidiales* 3–8*” provide a clear example (Fig. 10), as most of them were detected in a broad variety of thalli (e.g., thalli of *Enterographa*, *Dendrographa*, *Dirina*, *Roccella* or *Roccellographa*). In addition, *Cystobasidiomycetes* were not detected in all fruticose thalli; this is the case for *Roccella* in the present study (Suppl. material 4: table S1), and similarly in other lichens, where the yeasts were not detected in all specimens (Suppl. material 4: table S1; Spribille et al. 2016; Lendemer et al. 2019).

By contrast, some OTUs seem to be restricted to taxa with a single thallus type, particularly in the case of genus- or species-selective *Cystobasidiomycetes* associated with *Bryoria*, *Cladonia*, *Opegrapha* or *Syncesia* (Figs 3, 10, Suppl. materials 1, 2; Spribille et al. 2016; Černajová and Škaloud 2019; Nguyen et al. 2023; this study). In these cases, either sampling is insufficient and these OTUs are in reality associated with a broader range of mycobionts than currently observed, or they are truly selective for a particular lichenized species. However, such specificity does not necessarily imply a functional role in cortex or thallus development (e.g., *Opegrapha* species are crustose and ecorticate; *Syncesia myrticola* is corticate, but crustose). Our results corroborate and extend the observations of Černajová and Škaloud (2019). Whereas their study showed that cystobasidiomycete yeasts are not restricted to corticate species within *Cladonia*, our broader sampling across *Arthoniales* suggests that this pattern is not confined to a single genus but extends across a morphologically and phylogenetically diverse lichen lineage. However, it cannot be excluded that they may play other roles within the lichen microbiome, such as involvement in the synthesis of certain secondary metabolites, as highlighted for *Bryoria fremontii* (Spribille et al. 2016). Moreover, there may be particular yeasts lineages that may play cortex-associated functions, not necessarily related to the synthesis of the cortex itself.

However, differences in wet lab and sequencing methods may also lead to different outcomes. The study of Nguyen et al. (2023), for example, demonstrates it. In the latter, 15 specimens of *Cladonia rei* were used to obtain basidiomycete yeasts sequences by culturing and PCR, and for seven of them, a metabarcoding approach, with Illumina sequencing, was also used. For those seven specimens, yeasts were found in six of them with the metabarcoding approach, but only in three with the culturing method. PCR biases are also possible, potentially failing the detection of a target, especially when present in small quantities. In addition, our samples consisted of specimens with different thallus thicknesses, from very thin thalli (like in *Opegrapha*) to very thick thalli (like in *Dirina*), meaning that detection probability is not necessarily equivalent across our specimens. It is therefore important to take into account the methodology used to interpret results, and ideally, to use several methods in the same study for comparison, though this is not always possible.

## Conclusion and perspectives

The three main findings of this study are that (1) lichen-derived *Cystobasidiomycetes* were detected in only one third of our samples and did not seem to be ubiquitous; (2) that there is no correlation between their presence and cortex presence or thallus growth form; and (3) that lichen-derived OTUs belong largely to specific lineages, separated from OTUs detected from other sources. Moreover, some OTUs exhibit a degree of preference in the mycobiont choice. More importantly, this work substantially expands current knowledge of *Cystobasidiomycetes* associated with *Arthoniales* lichenized fungi, with the detection of 21 OTUs, which were not detected from other lichenized groups, with only two exceptions (OTUs “*Microsporomycetaceae* 36” and *Millanizyma cladoniophila*). No robust conclusions could be made concerning climate and substrate, due to a lack of data from tropical or other extra-temperate regions, but it is plausible that these two environmental variables (especially climate) are likely to have a greater effect than estimated here (Fig. 9). Additional saxicolous, terricolous, but especially foliicolous lichens, as well as sampling from tropical regions should therefore complete the *Cystobasidiomycetes* dataset to truly understand the presence or absence of a climatic or substrate pattern in this group of fungi. Moreover, other factors, such as the photobiont taxonomy should also be studied, as similar patterns were observed between them (Borgato et al. 2022; Mark et al. 2020; Kosecka et al. 2022) and *Cystobasidiomycetes*: for this purpose, data should be enriched with lichenized fungi associated with *Trentepohliales* algae or other mycobiont species which were not yet considered for a study on lichen-associated fungi. It would also be useful to deepen sampling in a target habitat to study the influence of local conditions on *Cystobasidiomycetes* distribution and compare several ecosystems, like littoral and inlands forests or rocks, with a similar protocol as Mark et al. (2020) or Borgato et al. (2022). In addition, any wet lab technique has its own biases, leading to false positive or false negative results. It would therefore be important to compare the methodology used in this study with other techniques, such as culturing, Illumina sequencing, or shotgun sequencing, to detect eventual differences in the outcome.

Finally, lichen-associated fungi have been known for more than 200 years, yet only photobionts were long considered true symbiotic partners. This view has since been revised (e.g., Arnold et al. 2009; Spribille et al. 2016; Fernández‐Mendoza et al. 2017; Smith et al. 2020; Cometto et al. 2022, 2023, 2024), with at least some of these fungi, belonging to several independent lineages (e.g. *Agaricostilbomycetes*, *Cystobasidiomycetes*, *Dothideomycetes*, *Eurotiomycetes*, *Leotiomycetes*, *Microbotryomycetes* or *Tremellomycetes*) now regarded as secondary facultative symbionts, especially when they are endolichenic, not showing symptoms on the mycobiont thallus (Harutyunyan et al. 2008; Cometto et al. 2023). The development of next-generation sequencing (NGS) techniques has also greatly accelerated research in this field over the past decade (e.g., Spribille et al. 2016; Lendemer et al. 2019; Mark et al. 2020; Cometto et al. 2022, 2023, 2024; Tagirdzhanova et al. 2024; Vondrák et al. 2024, 2025; Kirkhus et al. 2025; Root et al. 2025; this study), leading to the discovery of many new fungal lineages as it is also the case in the present study, using MinION sequencing approach.

This study focused exclusively on *Cystobasidiomycetes*, which are facultative symbionts, since they can be both lichenicolous and endolichenic (Diederich et al. 2022b; Millanes et al. 2025). However, they represent only one group among several lichen-associated fungal lineages. Future research should therefore also address other fungal classes associated with *Arthoniales*, with priority given to those most frequently detected and known to include lichen-associated taxa, such as *Tremellomycetes*, *Dothideomycetes*, and *Eurotiomycetes*.

## Additional information

### Artificial Intelligence (AI) use

The authors accept full responsibility for the content of the manuscript, including the disclosure of any use of AI.

Regarding the use of AI in the preparation of this manuscript, the authors declare the following: GPT

Used for: Language, style and writing

ChatGPT AI tools were used to improve English language and style for some sentences or paragraphs. It has also been used during the review process, especially for rewriting the discussion. Text generated by ChatGPT was not simply copy-pasted, but was subsequently adapted manually to make it fit in the manuscript context.

### Adherence to national and international regulations

Specimen collection was conducted by respecting the Nagoya protocol regulations, where applicable. Specimens collected in Belgium did not require a permit, since the research host institutions were situated in the same country. Specimens from Italy did not require a permit, since Italy did not adhere to Nagoya protocol.

### Author ORCIDs

L. Borgato https://orcid.org/0000-0001-5120-7253

P. Asselman https://orcid.org/0000-0003-3491-2174

D. Ertz https://orcid.org/0000-0001-8746-3187

M. Amalfi https://orcid.org/0000-0002-1792-7828

A. Verbeken https://orcid.org/0000-0002-6266-3091

### Data availability

DNA sequences generated in this study were deposited in GenBank (https://www.ncbi.nlm.nih.gov/genbank/). Additional figures and tables (Suppl. materials 1–4) are provided as supplementary material (see legends and links below).

## Supplementary materials

Supplementary material 1 Total consensus IQ-Tree of *Cystobasidiomycetes*, from ML analysis based on ITS data set with bootstrap support values ≥70% from Ultra-Fast Bootstrap analysis presented near the nodes Data type pdf https://binary.pensoft.net/file/1814804This dataset is made available under the Open Database License (http://opendatacommons.org/licenses/odbl/1.0/). The Open Database License (ODbL) is a license agreement intended to allow users to freely share, modify, and use this Dataset while maintaining this same freedom for others, provided that the original source and author(s) are credited. Luca Borgato, Pieter Asselman, Damien Ertz, Mario Amalfi, Annemieke Verbeken

Supplementary material 2 Cladogram showing *Cystobasidiomycetes* sequences from the phylogenetic tree and their clades, classified by OTU, with their source of origin Data type xlsx https://binary.pensoft.net/file/1814805This dataset is made available under the Open Database License (http://opendatacommons.org/licenses/odbl/1.0/). The Open Database License (ODbL) is a license agreement intended to allow users to freely share, modify, and use this Dataset while maintaining this same freedom for others, provided that the original source and author(s) are credited. Luca Borgato, Pieter Asselman, Damien Ertz, Mario Amalfi, Annemieke Verbeken

Supplementary material 3 Rarefaction curves Data type pdf https://binary.pensoft.net/file/1814806This dataset is made available under the Open Database License (http://opendatacommons.org/licenses/odbl/1.0/). The Open Database License (ODbL) is a license agreement intended to allow users to freely share, modify, and use this Dataset while maintaining this same freedom for others, provided that the original source and author(s) are credited. Luca Borgato, Pieter Asselman, Damien Ertz, Mario Amalfi, Annemieke Verbeken

Supplementary material 4 Additional tables Data type xlsx Explanation note **table S1**. List of specimens used in this study. **table S2**. *Cystobasidiomycetes* sequences obtained in this study. **table S3**. List of *Cystobasidiomycetes* ITS sequences retrieved from GenBank. **tables S4a–c**. Chimeric sequences, sequences wrongly identified as *Cystobasidiomycetes* and *Cystobasidiomycetes* sequences previously identified as other fungi found on GenBank. **table S5**. Number of principal components axes used in the PCA analyses. https://binary.pensoft.net/file/1814807This dataset is made available under the Open Database License (http://opendatacommons.org/licenses/odbl/1.0/). The Open Database License (ODbL) is a license agreement intended to allow users to freely share, modify, and use this Dataset while maintaining this same freedom for others, provided that the original source and author(s) are credited. Luca Borgato, Pieter Asselman, Damien Ertz, Mario Amalfi, Annemieke Verbeken
