# Supplementary material for: Amplicon sequencing reveals host-associated lineages of Cystobasidiomycetes (Basidiomycota, Pucciniomycotina) in Arthoniales (Ascomycota, Arthoniomycetes), but no association with thallus architecture
Source: IMA Fungus. 2026 Oct 21;17:e201170. doi: 10.3897/imafungus.17.201170 (PMC13717492; doi:10.3897/imafungus.17.201170)
Supplement: Supplementary file 1 [file imafungus-17-e201170-s001.pdf]

Figure S1. Total consensus IQ-Tree of *Cystobasidiomycetes*, from ML analysis based on ITS data set with bootstrap support values ≥70% from Ultra-Fast Bootstrap analysis presented near the nodes. Sequences from this study are in bold, and sequences from *Cystobasidiomycetes* derived from *Arthoniales* are marked with an asterisk (*). Sequences are coloured following the source of origin of the specimen (grey: unknown source; black: environment; red: animal; green: plant; brown: fungus; blue: lichen). For each sequence, the country, the climatic zone (see Table S3 for codes significations) and the host (where relevant) are given (when available). Clades refer to *Cystobasidiomycetes* orders. OTUs are not indicated here, but are represented in Figure S2.

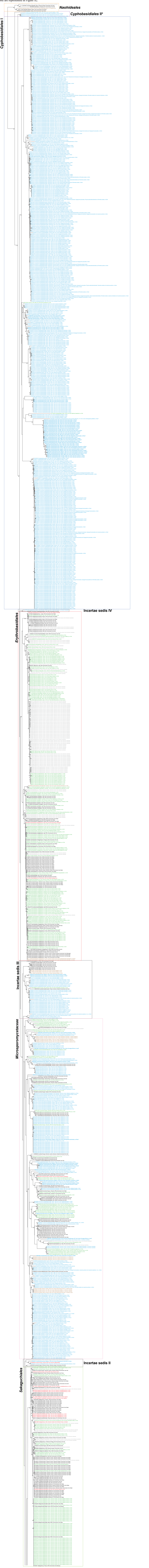

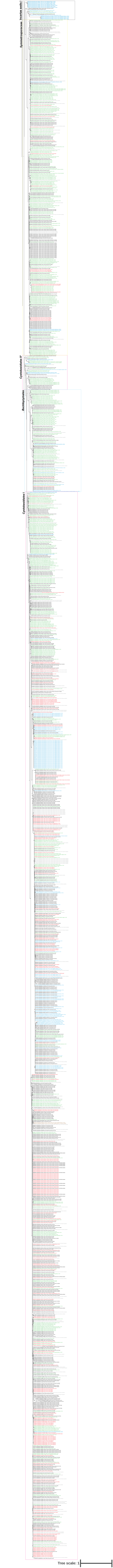

Incertae sedis I
Symmetrosporaceae
Cystobasidiales II
Buckleyzymales
Cystobasidiales I
Tree scale: 1
