# Supplementary material for: Amplicon sequencing reveals host-associated lineages of Cystobasidiomycetes (Basidiomycota, Pucciniomycotina) in Arthoniales (Ascomycota, Arthoniomycetes), but no association with thallus architecture
Source: IMA Fungus. 2026 Oct 21;17:e201170. doi: 10.3897/imafungus.17.201170 (PMC13717492; doi:10.3897/imafungus.17.201170)

Figure S3. (**a**) Rarefaction curves of the (**A**) *Buckleyzymales*, (**B**) *Cyphobasidiales*, (**C**) *Cystobasidiales* and (**D**) *Microsporomyceatceae* clades, which contain the majority of lichen-derived *Cystobasidiomycetes* sequences, showing the sampling coverage of mycobiont host. (**b**) Rarefaction curves on *Arthoniales*-derived *Cystobasidiomycetes* sequences from samples of this study. **A** = all samples; **B** = samples with corticate mycobionts; **C** = Samples with crustose corticate mycobionts; **D** = samples with crustose ecorticate mycobionts; **E** = samples with fruticose mycobionts.

(a)

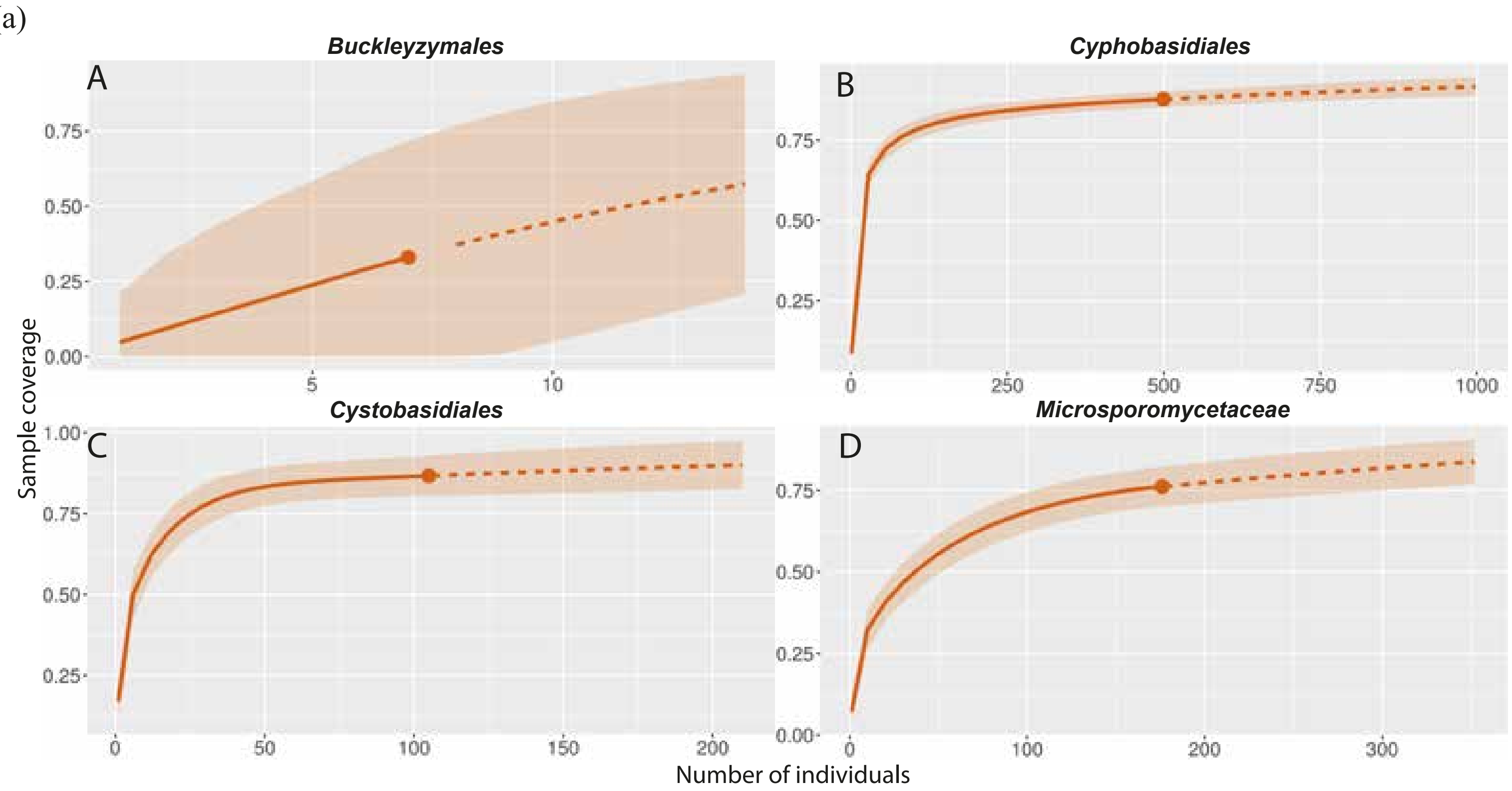

(b)

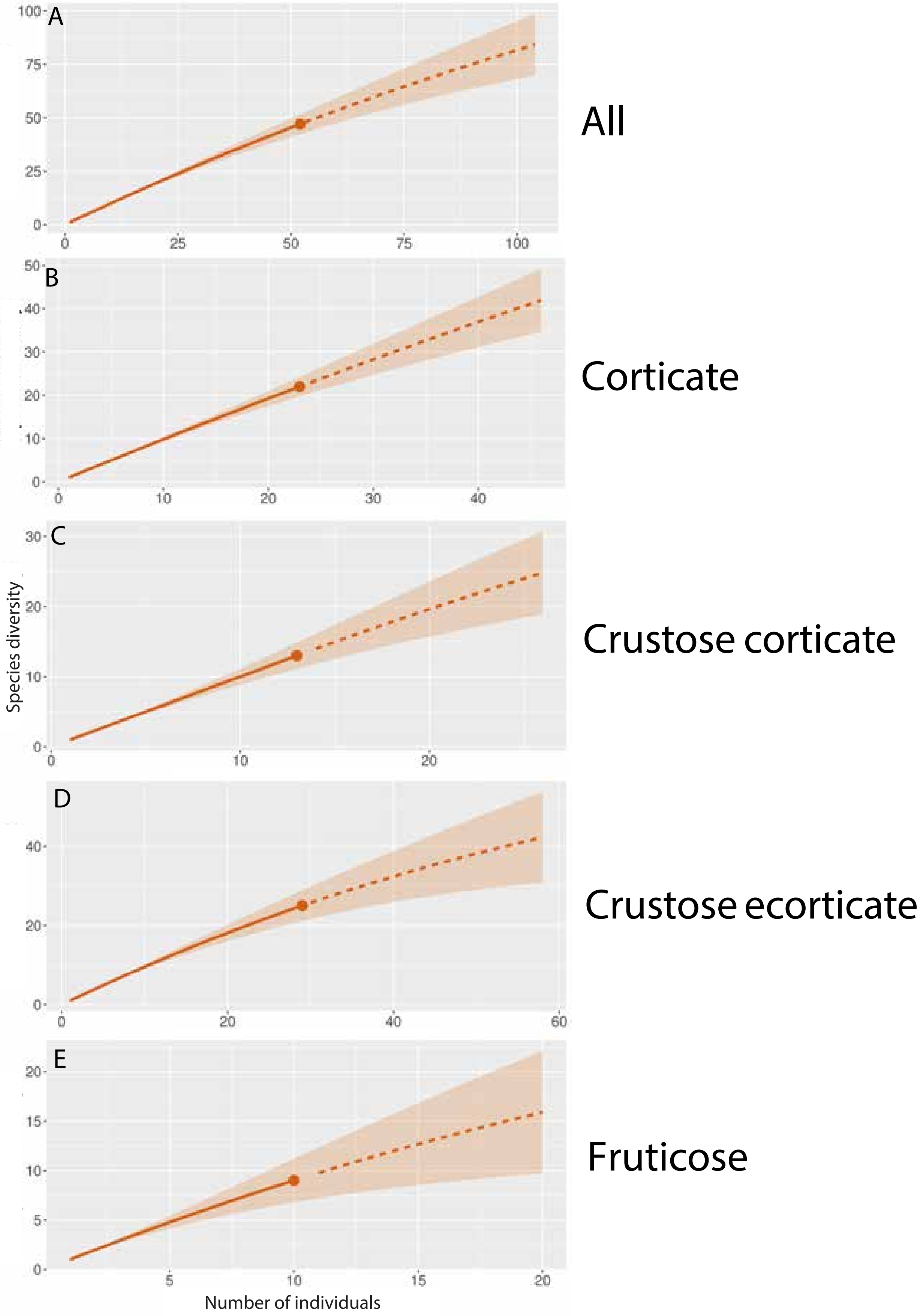

Supplement: Supplementary file 2 [file imafungus-17-e201170-s003.pdf]
